# Targeting glutamine metabolism as a potential target for cancer treatment

**DOI:** 10.1186/s13046-025-03430-7

**Published:** 2025-07-01

**Authors:** Wenxuan Zou, Zitao Han, Zihan Wang, Qian Liu

**Affiliations:** 1 Clinical Medicine Department, Xinjiang Medical University, Xinjiang, (New Medical Road Campus), No.393 Xinyi Road, Urumqi, 830054 China; 2 Department of Pathology, School of Basic Medical Sciences, Xinjiang Medical University, Xinjiang, (Xuelianshan Campus), No.567 Shangde North Road, Urumqi, 830011 China

**Keywords:** Glutamine metabolism, Malignant progression of tumors, Immune evasion, Anticancer targets, Drug resistance

## Abstract

Metabolic reprogramming is a hallmark of cancer cells, and the advent of “glutamine addiction” in numerous tumors signifies a pivotal advancement for precision-targeted therapy. This review demonstrates that glutamine metabolism is a pivotal factor in the development of malignant phenotypes in tumors by modulating multifaceted regulatory networks (Hippo/YAP, mTORC1 signaling pathway, and non-coding RNAs). These networks play a crucial role in the reprogramming of glutamine metabolism, which in turn affects various hallmarks of cancer, including cancer cell proliferation, ROS-mediated inhibition of apoptosis, and EMT-associated invasive metastasis. With respect to targeted therapeutic strategies, the focus on key transporters and metabolizing enzymes (ASCT2/GLS1) provides a theoretical foundation for the development of multi-targeted combination therapeutic regimens based on the inhibition of glutamine metabolism. A body of research has demonstrated that the metabolic processes of glutamine regulate a variety of immune system functions, including T cell depletion/activation, the polarization of TAMs, and the function of NK cells. This regulatory relationship, termed the metabolic-immune axis, is a crucial factor in the development of immune escape mechanisms by tumors. The study further suggests that a combination of targeted intervention strategies, involving the modulation of glutamine metabolism, has the potential to reshape the immune microenvironment and enhance the efficacy of CAR-T cell therapy. It is important to note that glutamine metabolism also affects tumor stroma formation by remodeling cancer-associated fibroblasts (CAFs). In response to therapeutic resistance mechanisms, tumor cells form adaptive escapes through ASNS and GAD metabolic branch activation, glucose/lipid metabolic compensation, and ATF4 transcriptional stress networks. This review systematically integrates the critical role of glutamine metabolism in tumor development and therapeutic resistance, providing new perspectives and translational pathways for the development of precision therapeutic strategy selection based on metabolic plasticity modulation.

## Introduction

Although the pathogenesis of tumours is complex, most tumours can meet their material and energy needs by altering cellular metabolic patterns. A comprehensive review of the therapeutic strategies employed in the fight against c-Myc reveals a multifaceted approach encompassing the following: the inhibition of its binding to DNA, the interference with its transcription, and the promotion of its degradation [1]. In 1924, Otto Warburg discovered that cancer cells exhibited a preference for glycolysis in the presence of sufficient oxygen, a phenomenon subsequently termed the Warburg effect [2]. In addition to the significance of glucose metabolism, the role of glutamine (Glutamine) in the tumour microenvironment is of particular interest. Glutamine provides carbon and nitrogen sources, thereby supplying energy to drive the tricarboxylic acid cycle (TCA) and support cancer cell growth [3, 4]. It is an irrefutable fact that cancer cells are unable to satisfy their glutamine requirement through endogenous synthesis alone; they must take up glutamine extracellularly to sustain themselves. Consequently, cancer cells are ‘glutamine addicted’ to glutamine [5]. In the preceding decade, translational therapies targeting cancer metabolism have seen only limited progress. A mere handful of drugs have been successfully developed and/or entered into clinical trials. In this review, we characterize glutamine metabolism in cancer cells and explore the specific mechanisms by which glutamine affects cancer cell proliferation, apoptosis, invasion and metastasis, and tumor immune escape. A synopsis and discourse on the carriers or enzymes associated with glutamine metabolism as novel “targets” that can regulate the state and development of cancer cells, as well as related clinical inhibitors, is warranted. However, further validation of their efficacy and safety through additional clinical trials is necessary. It is also emphasized that glutamine metabolism heterogeneity and adaptability become one of the main reasons for drug resistance in tumor patients, so we explore the mechanism of cancer cells utilizing glutamine metabolism-related branched pathways as well as bypassing glutamine metabolism to participate in the occurrence of drug resistance, which provides a new diagnostic and therapeutic idea for the further clinical development of glutamine metabolism-related antitumor drugs.

## Glutamine metabolism and cancer

Cancer cells have been observed to adapt to harsh environments, such as hypoxia and nutrient deficiencies, by reprogramming their metabolic pathways. This enables them to satisfy their energy needs and thus proliferate rapidly [6, 7]. Glutamine (Gln) is the most abundant amino acid in plasma in the body and provides a nitrogen and carbon source for the proliferation of cancer cells [8]. Eagle et al. were the first to discover a significantly higher demand for Gln for cancer cell proliferation when human HELA cells were cultured in vitro [9]. Since then, a large number of studies have shown that Gln addiction exists in many tumours, including cervical cancer, gastric cancer and colon cancer [10, 11]. Despite significant advancements in tumour treatment, the uncontrolled proliferation of cancer cells remains a primary factor contributing to the elevated recurrence rate and poor prognosis of tumour patients. Consequently, further elucidation of Gln metabolic profiles and the utilisation of carriers, enzymes, or metabolites during aberrant energy metabolism as potential targets for cancer therapy would be expected to improve patient prognosis.

**Fig. 1:**
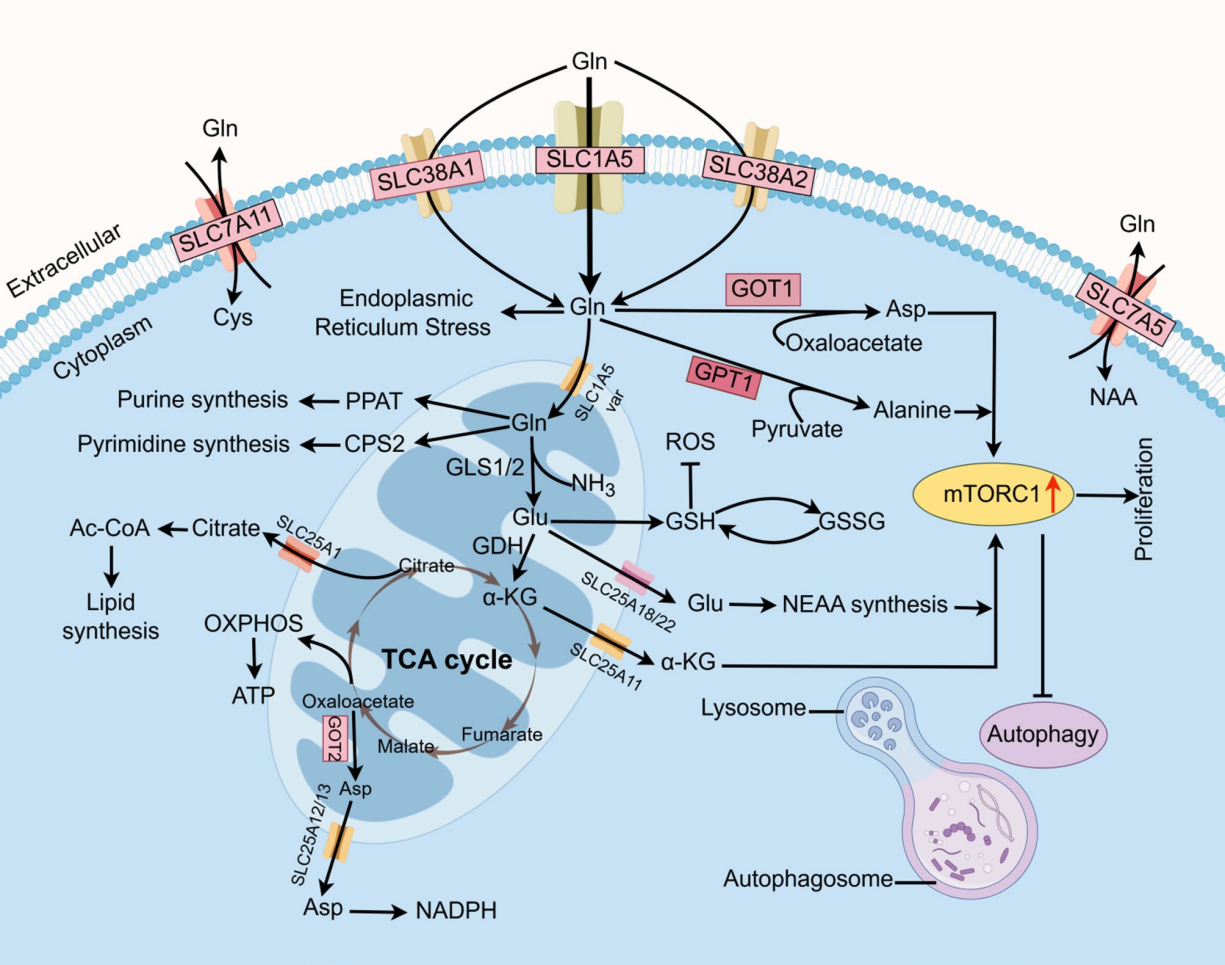
Glutamine metabolism in cancer cells. Gln enters cancer cells through transporter proteins such as SLC38A1, SLC38A2 and SLC1A5 (also known as ASCT2), and is converted to glutamate by the action of mitochondrial GLS 1/2, which is further converted to α-KG by GDH to enter the TCA cycle, which provides cancer cells with energy. Glutathione (GLULH) metabolism is a process that leads to the synthesis of GLULH, NADPH and other important anti-oxidative stress substances. These substances can resist intracellular reactive oxygen species (ROS) production. Finally, Gln can also be involved in lipid metabolism and amino acid metabolism, further enriching the amino acid pool via membrane carrier (SLC7A11), and co-activating mTORC1 with α-KG to inhibit cancer cell autophagy and promote cancer cell proliferation

The process of Gln entering the cytoplasm is primarily facilitated by a specific solute carrier, designated as ASCT2. After this, Gln is catabolised to glutamic acid (Glu) in the presence of glutaminase (GLS), which is identified as the rate-limiting step in the catabolism of Gln [12, 13]. This process is further catalysed by the action of mitochondrial glutamine dehydrogenase (GDH), resulting in the conversion to α-ketoglutarate (α-KG). The subsequent entry of α-KG into the tricarboxylic acid cycle (TCA cycle) is a pivotal step in providing energy for the proliferation of cancer cells (Fig. 1). In mammals, GLS is present as two isoenzymes: GLS1 (renal-type glutaminase) and GLS2 (hepatic-type glutaminase). These isoenzymes play key roles in regulating tumour cell growth, proliferation, metastasis, apoptosis and drug resistance. A plethora of studies have demonstrated that GLS1 demonstrates elevated expression in a multitude of tumours, including hepatocellular carcinoma (HCC) and prostate cancer (PCa) [14–16]. Furthermore, GLS1 has been associated with a poor patient prognosis. In the event of glutamate deprivation or GLS1 inhibition, there is a concomitant reduction in cell viability and elevation in G1 phase. Conversely, clinical studies have demonstrated that GLS1 promotes mitochondrial respiration rate and tumour growth potential [17]. Consequently, the mechanism of GLS1 action is more clearly defined, and the targeted inhibition of GLS1 has potential for use in cancer therapy. GLS2 exhibits a distinctly divergent expression pattern in comparison to GLS1, which functions as an environmentally dependent tumour suppressor gene and is associated with a favourable prognosis [18]. Nevertheless, evidence has emerged demonstrating that GLS2 is overexpressed in certain tumours, thereby contributing to their development. Therefore, the precise mechanism of action of GLS2 remains to be elucidated, and there is heterogeneity between different tumours. The function of GLS2 as either a tumour suppressor or an oncogene is contingent upon the specific type of tumour in question.

Gln metabolism has been demonstrated to play a significant role in tumour development. A comprehensive understanding of glutamine metabolism can facilitate a more precise estimation of the potential anticancer targets and offer valuable clinical guidance.

## The role of glutamine metabolism in the biological behaviour of cancer cells

### Glutamine metabolism as ‘fuel’ for cancer cell proliferation

Glutamine plays an important role in cell growth and proliferation. The dependence on glutamine has been identified as a hallmark of elevated cancer cell proliferation [19]. Gln metabolism supplies cancer cells with essential nutrients, including proteins, nucleic acids, and lipids, thus facilitating their proliferation. Glu, a product of metabolism, serves as a precursor for various non-essential amino acids, including alanine (Ala) and aspartic acid (Asp). The process of transamination within the body facilitates the synthesis of substantial amino acid pools, thereby contributing to the availability of essential protein sources. As Gln is known to provide γ-amino nitrogen, it is involved in the synthesis of purines and pyrimidines [20]. Recent studies have indicated that the glutamine (Gln) metabolite α-ketoglutarate (α-KG) can undergo conversion to acetyl-CoA, a process that plays an integral role in the metabolic process of fatty acids. Consequently, elevated ASCT2 expression levels were found to synergistically regulate PPARα expression levels and enhance fatty acid metabolism in a variety of tumors [21, 22]. Concurrently, it is noteworthy that extracellular fatty acids (FAs) can enhance Gln metabolism levels and promote tumor cell proliferation. Recent studies have identified a novel mechanism through which oleic acid (OA) promotes cancer cell proliferation. Specifically, OA enhances the uptake of glutamine by ovarian cancer cells and activates DNA synthesis in these cells, contributing to the proliferation of cancerous cells [23]. Research has indicated that SREBP-1 can upregulate the expression of ASCT2, which is responsible for the release of ammonia using promoting glutamine metabolism. Furthermore, ammonia has been observed to activate SREBP-1, thus establishing a feed-forward loop that promotes glutamine metabolism and lipid synthesis, as well as tumor cell proliferation [24]. The induction of mitochondrial damage and oxidative stress through the combined use of an inhibitor of glutamine metabolism and an inhibitor of lipid metabolism (pimozide) resulted in GBM cell death. Consequently, the further inhibition of Gln metabolism and fatty acid metabolism through the suppression of ASCT2 expression, which poses a significant threat to the energy supply required for cancer cell proliferation, emerges as a promising anticancer target.

**Fig. 2:**
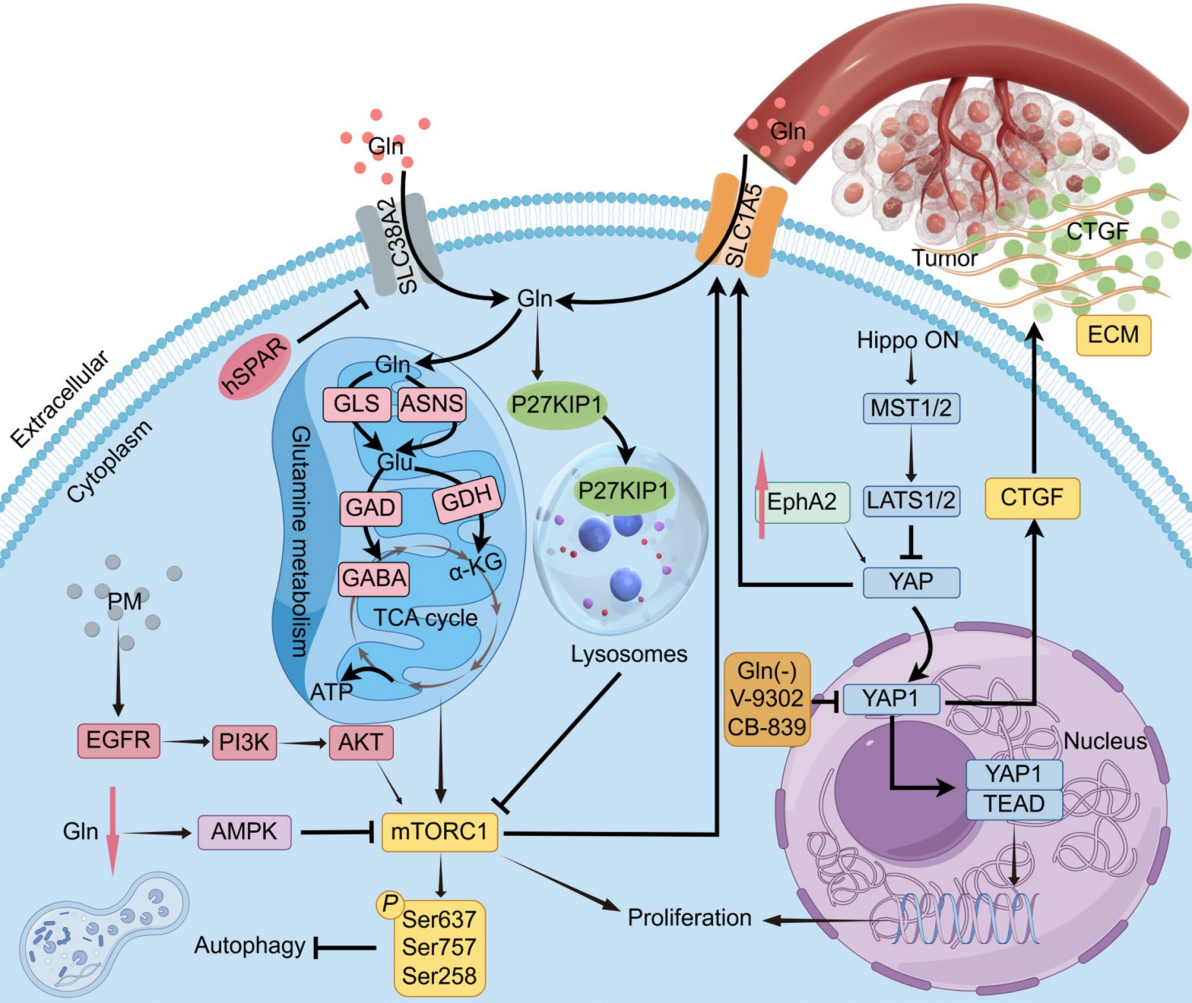
Gln modulates multiple signalling pathways to regulate cancer cell proliferation. In the event of Gln deficiency or Gln metabolism inhibition, it can activate YAP protein in the Hippo signaling pathway, and also inhibit cancer cell proliferation by inhibiting YAP entry into the nucleus and binding to TEAD. In addition, it has been demonstrated that YAP1 can promote ECM deposition by enhancing CTGF secretion and release, as well as ASCT2 expression, enhanced Gln hydrolysis, and activation of the mTOR1 signalling pathway to promote cancer cell proliferation. The AMPK-MTORC1 signalling axis has been identified as a key regulator, and GLS and ASNS have been shown to compensate each other for their respective activities, which has been identified as a significant factor contributing to the low anticancer efficiency observed when GLS inhibitors are used in isolation

It has been established that Gln exerts its regulatory function by modulating multiple signalling pathways, thereby controlling the proliferation of cancer cells using complex mechanisms (Fig. 2). The Hippo/YAP pathway is a signalling network that plays a critical role in regulating cell growth and proliferation in multicellular organisms. Adhikary G. et al. conducted a study on mesothelioma, also found that treatment of mesothelioma cells in culture medium with Gln deletion and depletion, the Gln uptake inhibitor V-9302, or the GLS inhibitor CB-839 significantly reduced mesothelioma cell growth by decreasing the level of expression of YAP1, the level of expression of YAP1/TEAD and its target proteins [25]. Park et al. discovered that the metabolism of glutamine can activate Yes-associated protein (YAP) in cancer cells by decreasing the levels of cyclic adenosine monophosphate (cAMP)/protein kinase A (PKA) phosphorylation in LATS [26]. The activation of YAP subsequently induces extracellular matrix (ECM) deposition through enhanced secretion of connective tissue growth factor (CTGF), while concurrently promoting the production of fibrous collagen and connective tissue by the surrounding fibroblasts. Consequently, the targeting of the Hippo/YAP pathway in conjunction with Gln metabolism inhibition may present a viable therapeutic approach for addressing tumors. Furthermore, enhanced glutamine metabolism has been shown to significantly increase α-KG production, which, in turn, activates the mTOR1 signaling pathway and further inhibits the autophagy-promoting kinase (APCK) of the ULK1 complex by mediating site-specific phosphorylation of ULK1 (Ser637 and Ser757) and Atg13 (Ser258) activity, thereby promoting cancer cell proliferation [27]. Human micropeptide (hSPAR) has been shown to inhibit the expression of the glutamine transporter protein SLC38A2, thereby decreasing Gln levels in cancer cells. This, in turn, has been observed to trigger a series of events that result in the translocation of cytoplasmic P27KIP1 to the lysosome. Disruption of the Ragulator complex, inhibition of the assembly of the mTORC1 complex, and inhibition of the proliferation of breast cancer cells have also been identified as sequelae of this process [28]. Recent findings have indicated that the mTOR signaling pathway functions as an upstream regulator of glutamine metabolism, thereby modulating cellular proliferation. A subsequent RNA sequencing analysis revealed that amphiphysin (AREG) plays a pivotal role in particulate matter (PM)-induced lung cancer cell proliferation. Furthermore, PM is be involved in SLC1A5 expression and glutamine metabolism through the EGFR/PI3K/AKT/mTOR signaling pathway, thereby promoting the proliferation of lung cancer cells [29]. Furthermore, the AMPK-MTORC1 signaling axis functions as a pivotal regulator of cell growth and proliferation [30]. It has been determined that when glutamine depletion activates the energy stress AMPK pathway and inhibits mTORC1 activity, this results in the subsequent inhibition of the protein expression level of β-TrCP, consequently leading to abnormal cell cycle progression and reduced proliferation [19].

The process of glycolysis can be subject to modulation by a variety of oncogenes, which in turn can impact the proliferation of cancer cells. In patients with KRAS-mutant human colorectal cancer (CRC), the expression of the transcription factor, YAP1, has been observed to promote the expression of the SLC1A5/ASCT2 gene, which is involved in amino acid uptake. This enhanced uptake of amino acids has been shown to further activate the mTOR signaling pathway, thereby promoting the proliferation of CRC cells [31]. In a similar manner, an increase in the expression of GLS1 and ASCT2 was observed in ovarian cancer cells with a KRAS mutation. This upregulation led to an augmentation in glutamine metabolism and a subsequent promotion of cell proliferation [32]. Mitochondrial ribosomal protein L35 (MRPL35) is found to be highly expressed in non-small cell lung cancer (NSCLC) cells, and the anticancer effect of MRPL35 silencing can be rescued by promoting SLC7A5 expression [33]. In HER2-positive breast cancer, the overactivation of the receptor tyrosine kinase EphA2 has been demonstrated to induce the expression of the transcription factors YAP/TAZ and their downstream target genes, thereby promoting cell proliferation [34]. TAR (HIV-1) RNA-binding protein 1 (TARBP1), which also functions as an oncogene, has been shown to promote ASCT2 expression and Gln input by selectively methylating and stabilizing a small portion of tRNA to drive cancer cell growth [35]. Cell migration-induced hyaluronan-binding protein (CEMIP), an oncogenic protein, is highly expressed in small cell lung cancer (SCLC) and promotes proliferation by increasing glutamine depletion and glutamate and glutathione levels in SCLC cells [36]. A body of research in the epidemiological sciences has demonstrated that a mitochondrial enzyme, which is responsible for encoding a bifunctional mitochondrial enzyme known as ALDH18A1, exhibits elevated levels of expression. This heightened expression has been observed to result in a substantial reduction in patient survival, a phenomenon that is attributed to the enzyme’s capacity to enhance glutamine metabolism within cancerous cells. Consequently, ALDH18A1 is regarded as a pivotal gene with the potential to significantly impact risk models, underscoring its importance in clinical and health-related decision-making processes [37]. The study revealed that the oncogene SIRT4 exerts its inhibitory effect on glutamine metabolism, thereby suppressing the development of cervical cancer through the MEK/ERK/C-MYC signaling pathway. This finding provides a novel framework for the development of novel therapeutic interventions for cervical cancer [38]. The aforementioned study establishes a theoretical foundation for the development of tumor molecular precision targeting in combination with glutamine metabolism-inhibiting drugs. This approach holds significant value in facilitating the development of more precise and personalized treatment plans, thereby enhancing the prognosis of tumor patients.

The potential role of the ubiquitin-proteasome system (UPS) in the regulation of glutamine metabolism in tumor cells remains to be fully elucidated. Consequently, elucidating the function of ubiquitination and deubiquitination modifications on glutamine metabolizing enzymes in tumor cells is anticipated to yield novel concepts for the formulation of novel clinical treatment strategies for tumor patients. USP47, a deubiquitinating enzyme, is overexpressed in cancer cells, and its binding to U box-containing protein 1 (SATB1) promotes its deubiquitination level and thus glutamine metabolism. These findings suggest that targeting the USP47/SATB1 signaling axis to inhibit PTC glutamine metabolism is a promising therapeutic strategy [39]. Research has demonstrated that glutamate dehydrogenase (GLUD1) is expressed at a high level in lung adenocarcinoma cells, and lysine 503 (K503) has been identified as the primary ubiquitination site of GLUD1. Inhibition of the ubiquitination of this site has been demonstrated to promote the proliferation of lung adenocarcinoma cells and the growth of tumors. This provides a theoretical basis for the development of anticancer drugs targeting GLUD1 [40].

Long-stranded non-coding RNAs (lncRNAs) have been shown to play a crucial role in the regulation of cancer cell proliferation. The lncRNA FERRIN, which is produced under conditions of glutamine starvation induced by the transcription factor ATF4, interacts with the RNA-binding protein hnRNPK, thereby promoting its binding to and stabilizing SLC7A11, and consequently promoting the proliferation of cancer cells [41]. Furthermore, evidence has emerged demonstrating that LINC01764 exhibits a specific binding affinity for hnRNPK, thereby promoting its interaction with the oncogene c-MYC and facilitating the translation of ribosomes within c-MYC. This process culminates in the exertion of an oncogenic effect, the enhancement of glutamine metabolism, and the promotion of CRC cell proliferation [42]. The RNA cytosine-C(5)-methyltransferase (NSUN2) is found to be up-regulated in gastric cancer (GC), and the NR_033928, which acts as an up-regulated NSUN2 long non-coding RNA (lncRNA) to promote GLS expression by interacting with the IGF2BP3/HUR complex, is closely associated with the development of gastric cancer, the progression of the disease, and a poor patient prognosis [10].

A substantial body of research has emerged in recent years, focusing on the role of N6-methyladenine (m6A) RNA modification in glucose, amino acid, and lipid metabolism. This modification, being the most prevalent form of mRNA modification in eukaryotes, has emerged as a pivotal focus in biomedical research. The function of m6A is to regulate RNA interactions by determining their fate, thereby influencing a wide array of biological processes. A complex network of the “m6A-metabolite-TME” axis has been proposed, i.e., the ability of m6A to induce aberrant changes in metabolite levels, which may in turn trigger oncogenic signaling pathways leading to significant alterations within the TME [43]. Recent findings have indicated that levels of the atypical methyltransferase METTL1 are elevated in cancer cells, thereby promoting glutamine synthase (GLUL) expression in an m6A-dependent manner, which in turn facilitates cancer cell proliferation [44]. In the future, there is still a need for systematic in-depth (m6A) RNA modification mechanisms and metabolic function-related studies. These studies will be important for the development of novel and targeted therapeutic strategies in cancer research.

### Glutamine metabolism inhibits apoptosis

The deprivation of Gln, or the obstruction of the glutamine hydrolysis pathway, has been demonstrated to promote apoptosis in cancer cells. This process can be achieved through a variety of mechanisms, and the apoptotic pathways induced exhibit significant differences between cell types.

Typically, cancer cells encounter elevated levels of oxidative stress during the proliferation phase. However, this does not typically result in their death due to the capacity of Glu to synthesize glutathione (GSH), a vital antioxidant that plays a crucial role in cancer cells and their resistance to oxidative stress [45]. Recent studies have demonstrated that in triple-negative breast cancer (TNBC), a substantial decrease in cancer cell GSH levels and a significant increase in cellular superoxide levels can be induced by the administration of a dual metabolic inhibitor of GLS and xCT (SLC7A11), which promotes apoptosis [46]. Similarly, when Gln is absent and the Gln hydrolysis pathway is blocked, a significant amount of reactive oxygen species (ROS) is produced in cancer cells. ROS can mediate the onset of cellular death, including conventional apoptosis (i.e., the mitochondrial pathway, the death receptor pathway, and the endoplasmic reticulum pathway), cellular scorched death, and iron death. The role of ROS in mediating mitochondrial apoptosis was investigated, and the results indicated that the inhibitor RX108 significantly downregulated the expression of ASCT2. This, in turn, led to a substantial reduction in energy metabolism in Huh7 and Hep3B cells. The reduction in energy metabolism was accompanied by lower levels of GSH, NADH, NADPH, and the rate of mitochondrial respiratory oxygen consumption. The accumulation of ROS was found to promote apoptosis in cancer cells [47]. Recent findings have indicated that the inhibition of glutamine metabolism can lead to a suppression of cancer cell apoptosis, a process that is believed to be influenced by the level of reactive oxygen species (ROS) metabolism. Zhao et al. found that GLUD1 is highly expressed in hepatocellular carcinoma, which enhances intracellular mitochondrial respiratory activity and generates large amounts of ROS. The excess of ROS leads to increased expression levels of apoptosis-promoting factors p53, Cytc, Bax, and Caspase3. The expression level of the apoptosis inhibitor Bcl-2 was reduced [48]. Concurrently, certain inhibitors have been identified as effective agents in the attenuation of ROS-mediated mitochondrial apoptosis. These inhibitors function by impeding the expression of ASCT2, a process exemplified by the action of lobetyolin. This compound has been observed to curtail ROS accumulation, thereby diminishing mitochondrial-mediated apoptosis. This effect is achieved through the downregulation of ASCT2 expression in MKN-45 and MKN-28 cells [49]. Consequently, the role of ROS in tumor progression is multifaceted, exhibiting both beneficial and detrimental effects. In addition, ROS can mediate cellular pyroptosis. The latest research shows that nanoparticles assembled from the ASCT2 inhibitor V-9302 and the glucose transporter GLUT1 inhibitor BAY-876 can inhibit the uptake of Gln and glucose by pancreatic cancer (PC) cells, leading to increased nutrient deficiency and oxidative stress. The ROS family produced significantly by oxidative stress can activate Caspase1 and GSDMD, accompanied by the release of a large number of pro-inflammatory factors, thereby inducing cell pyroptosis. This mechanism has also been confirmed in uveal melanoma (UVM) [50, 51]. ROS have been shown to induce cellular iron death simultaneously. Cai et al. conducted a study that revealed the accumulation of α-KG within cells to be a catalyst for the promotion of oxidative stress in lymphoma cells, resulting in the generation of substantial quantities of ROS, elevated levels in ROS promote cellular iron death by instigating lipid peroxidation and TP53 activation [52]. The utilisation of inhibitors of glutamine metabolism has been demonstrated to promote ROS formation and desensitization in PC cells, consequently resulting in altered cytokine-cytokine receptor interaction pathways, including those involving CCL5, CCR4, LTA, CXCR4, IL-6R and IL-7R, which in turn promote iron death [53]. As stated in, the treatment of oral squamous cell carcinoma (OSCC) with iron death inducer (PL) has been shown to result in lipid peroxidation (LPO) and intracellular ROS accumulation, accompanied by a decrease in the expression of FTH1, SLC7A11, and GPX4. The combination of PL and the glutaminase inhibitor CB-839 has been shown to exhibit a synergistic effect, resulting in a significant reduction in cell viability and levels of lipid peroxidation (LPO). This effect is accompanied by substantial depletion of glutathione (GSH) [54]. Therefore, CB-839 has been demonstrated to enhance PL-induced iron death synergistically. The synergistic anticancer effect of these two agents has the potential to serve as a novel strategy for cancer therapy. Evidence suggests that glutamine deficiency currently regulates the occurrence of multiple death pathways. However, further investigation is required to determine whether it also mediates copper-induced cell death. Moreover, it has been demonstrated that glutamine deprivation can mediate the TRAIL pathway in death receptor apoptosis. Gln depletion treatment activates FADD and caspase-8-dependent apoptosis involving pro-apoptotic TNF-related apoptosis-inducing ligand receptor 2 (TRAIL-R2) in tumour cells, while metabolic stress induced by glutamine deprivation leads to the downregulation of the GCN2-non-dependent FLICE-inhibitory protein (FLIP), which promotes caspase-8 activation and apoptosis [55].

Several drugs have been demonstrated to regulate apoptosis in cancer cells by modulating glutamine metabolism and thereby regulating apoptosis. Gefitinib has been shown to enhance glutamine catabolism in A549 cells, thereby accelerating apoptosis in the NSCLC cell line A549. This effect is achieved by promoting GDH1 expression through the downregulation of the epidermal growth factor receptor (EGFR)/MEK/ERK signaling pathway [56]. Furthermore, baicalein has been shown to inhibit the mTOR signaling pathway and induce apoptosis by down-regulating glutamine metabolism [57]. Silibinin has been shown to regulate the YY1/SLC1A5 axis, thereby promoting apoptosis in GBM cells [58]. The treatment of hepatocellular carcinoma with N-acetylcysteine (NAC) has been shown to impede GLUD1 activation and mitochondrial apoptosis via the ROS-mediated p38/JNK MAPK pathway [48].

Therefore, elucidating the relationship between glutamine metabolism and apoptosis, as well as the molecular mechanism, is imperative. Furthermore, exploring the potential therapeutic targets in a multi-dimensional manner can provide a theoretical foundation for clinical treatment.

### Glutamine metabolism and cancer cell invasion and metastasis

Despite the grave nature of primary tumours, it is important to note that they are responsible for only 10% of tumour-related fatalities. The remaining 90% of patients succumb to metastatic tumours, which develop at sites other than the primary foci. Therefore, invasive metastasis in the late stage of a tumour represents a critical and urgent problem that must be addressed in the study of tumour pathogenesis. The development of tumours is largely dependent on complex biochemical alterations in the tumour cells themselves and the cellular stroma. These are combined with varying degrees of glutamine dependence in cancer cells. In this section, an attempt is made to elucidate the signalling pathways and associated mechanisms that underpin the association between cancer cell glutamine metabolism and cancer cell adhesion, invasive movement and migration.

The invasive-metastatic cascade is a complex process involving multiple genes that regulate glutamine metabolism, thereby influencing the invasive-metastatic ability of cancer cells. In their bioinformatics analysis of the Cancer Genome Atlas Head and Neck Squamous Cell Carcinoma (HNSCC), Yang et al. found that the MYC gene, which codes for the protein c-MYC, acted as an upstream gene. This was due to its ability to bind directly to the GLS1 promoter region, thereby promoting GLS1 expression [59]. Furthermore, disruption of GLS1 signalling using gene depletion or the use of the Gln inhibitor CB-839 will also lead to ubiquitination of c-MYC proteins, creating a GLS1-c-MYC positive feedback loop. This positive feedback loop is a key driver of invasive metastasis in HNSCC by augmenting CoA carboxylase-dependent Slug acetylation. The development of specific inhibitors of these enzymes would be a very promising therapeutic strategy. Dysregulation of S100 calcium-binding protein A2 (S100A2) has been implicated in the development of several cancers. The present study investigates the relationship between the transcriptional regulator transcription factor AP-2 alpha(TFAP2A) and S100A2, both of which have been observed to show high expression in lung adenocarcinoma (LUAD) [60]. The silencing of TFAP2A has been demonstrated to inhibit glutamine metabolism and cellular translocation. However, this process is reversible, with the upregulation of S100A2 having the capacity to reverse the effects of TFAP2A silencing [60]. The TFAP2A-S100A2 axis has been demonstrated to promote LUAD metastasis by regulating glutamine metabolism. Tandem C2 structural domain nuclear protein (TC2N) is associated with tumorigenesis. TC2N has been demonstrated to activate the Wnt signalling pathway, thus promoting the invasive metastasis of HCC cells. This process is achieved by regulating the expression levels of β-catenin and its downstream targets, including CyclinD1, MMP7, c-Myc, c-Jun, AXIN2 and glutamine synthase [61]. The central gene actin-related protein 2/3 complex subunit 1 A (ARPC1A) is highly expressed in PCa, and it was found that glutamine metabolism promotes migration, invasion, and cytoskeletal changes in PCa cells through ARPC1A [62]. Consequently, the continuous excavation of genes upstream and downstream of glutamine metabolism, which can influence cancer cell invasion and metastasis-related genes, will be a promising means to contribute to the design of clinically specific relevant inhibitors.

The process of epithelial-mesenchymal transition (EMT) has been demonstrated to be a significant factor in the promotion of cancer cell invasion and metastasis. A recent study has revealed that solute carrier family 38 member 3 (SLC38A3) exerts a regulatory effect on the activity of glycogen synthase kinase 3-β (Gsk3β), a negative regulator of β-catenin. The findings indicate that SLC38A3 functions as an inhibitor of Gsk3β, leading to an increase in the protein level of β-catenin. This, in turn, results in the upregulation of EMT-inducible transcription factors and EMT markers in breast cancer. Furthermore, SLC38A3 has been observed to promote the metastasis of breast cancer [63]. Glutamine synthetase (GLUL) is a pivotal enzyme that catalyses the conversion of ammonium and glutamate to Gln in an endogenous synthetic process. The expression level of GLUL was found to be significantly lower in cancer tissues. Furthermore, the knockdown of GLUL was found to promote cancer cell growth and invasive metastasis in vitro and in vivo [64]. GLUL is known to compete with β-catenin for binding to N-calmodulin. This results in an increase in the stability of N-calmodulin and a decrease in the stability of β-catenin through the process of ubiquitination. The expression levels of N-calmodulin and β-catenin in cancer tissues have been observed to be low and high, respectively. Conversely, GLUL stabilises N-calmodulin by antagonising β-catenin, thereby impeding cancer invasion and metastasis. GLUL is highly expressed in HCC cells, and it has been demonstrated that this mediates EMT, thus promoting HCC cell invasion and metastasis [65]. Curcumin-inducible HIF-1α degradation has been shown to impede the process of invasive metastasis in colorectal cancer cells by impeding glutamine catabolism through the inhibition of GLS1 and the suppression of EMT [66]. Consequently, the inhibition of EMT emerges as a promising therapeutic strategy. Furthermore, EMT is induced by metabolic stress, which promotes the invasive metastasis of cancer cells. It has been determined that the deprivation of glutamine can induce metabolic stress, which in turn can result in the expression of ZNFX1 antisense RNA1 (ZFAS1). ZFAS1 has been shown to enhance the interaction between the key kinase AMPK and the major regulator of EMT, ZEB1. This enhanced interaction leads to the phosphorylation of ZEB1 and further stimulation of pancreatic cancer (PC) EMT and metastasis [67].

The glutamine transporter protein family (SLC) is localised on the cell membrane and enables cancer cells to take up sufficient Gln from the outside to meet their growth and metabolic requirements. Its expression also promotes tumour growth and metastasis and reduces apoptosis and oxidative stress through certain signalling pathways. The SLC38A3 (SNAT3) gene is known to be overexpressed in cases of triple-negative BC and has been shown to promote cancer cell invasion and metastasis through the SLC38A3/GLULK3β/β-linker protein/EMT pathway [63]. SLC1A5 antisense long non-coding RNA (SLC1A5-AS) has been observed to be highly expressed in cases of HCC, enhanced Gln uptake and association with poor prognosis in HCC. It has been established that elevated levels of SLC1A5-AS bind directly to myeloid zinc finger 1 (MZF1), thereby acting as a transcriptional deterrent and reducing its binding to the SLC1A5 promoter region. This process significantly promotes the invasive metastasis of HCC cells [68]. Consequently, the targeting of SLC1A5-AS/MZF1 in combination with ASCT2 inhibitor therapy may represent a viable strategy for the treatment of HCC.

In the 1950s, the scientific community identified non-coding RNAs (ncRNAs) as playing a pivotal role in gene expression. This categorisation encompasses ribosomal RNA (rRNA) and transfer RNA (tRNA), which are defined as functional RNA molecules that do not undergo translation into proteins. In the decades that followed, as a result of the growing understanding of tumours, classical ncRNAs were the focus of extensive research. In the decades that followed, as a result of the growing understanding of tumours, classical ncRNAs were the focus of extensive research. The differential expression of circular RNAs (circRNAs) and microRNAs (miRNAs) in cancer is a useful predictor of their potential role in cancer progression, whether as oncogenes or tumour suppressors [69]. CircRNAs are a class of non-coding RNA molecules characterised by their ability to maintain stability and be expressed at high levels in a closed-loop structure. These molecules are found in abundance in eukaryotic cells and have been identified as playing a critical role in tumour metabolism in various cancers. Additionally, they have been observed to regulate the expression of miRNAs, further highlighting their importance in cellular processes. It has been demonstrated that miRNAs are closely related to cancer cell invasion and metastasis. However, it is also evident that they can interfere with several metabolic processes that lead to the regulation of cancer cell motility. In addition, it has been established that they connect metabolic and adhesion/migration signalling pathways, thus altering the spreading and metastasis formation of cancer cells that usually affect them [70]. Recent studies have revealed a novel finding: miRNAs and circRNAs exhibit antagonistic behaviour in different types of cancer tissues. Collectively, these molecules form a regulatory network that impacts the invasive and migratory capabilities of cancer cells, in conjunction with the Glucose-Limiting Sensor (GLS). Qian et al. found that Circ_00010993 was highly expressed in esophageal squamous cell carcinoma (ESCC) and increased GLS expression by adsorption of microRNA-579-3p. Concurrently, the expression levels of microRNA-579-3p in ESCC tissues were found to be low, while those of GLS were found to be high. This further promoted cancer cell invasion and metastasis [71]. It has been established that Circ_0001093 and miR-579-3p are in mutual negative feedback, and that, in conjunction with GLS, the three form the Circ_0001093/miR-579-3p/GLS regulatory network. This network can influence invasive metastasis in ESCC. This phenomenon has also been evidenced in melanoma and CRC [72, 73]. As demonstrated in (Table 1), the mutual negative regulatory effects of circRNAs and miRNAs in different types of cancer tissues are listed. These regulatory effects are demonstrated to co-regulate the downstream glutamine metabolism targets and to show their effects on cancer cell invasion and metastasis. In the future, by continuously and deeply exploring the role of ncRNAs in the invasive movement of tumours and clarifying new molecular mechanisms in various types of cancers, it will help to discover new anti-cancer targets, biomarker molecules, develop new reagents and drugs, and provide new ideas for clinical diagnosis and treatment.

**Table 1:** Targets of glutamine metabolism downstream of CircRNA/miRNA and implications for invasive metastasis

CirRNA/miRNA Negative Feedback Regulatory Axis	Direct Target	Cancer Type	Effect On Cancer Cell Invasion And Metastasis	Refs
CirB3GNTL1/miR-598	—	Gastric cancer	↓	[74]
Circ-CREBBP/miR-375	GLS	Glioma cancer	↑	[75]
Circ_00000003/miR-330-3p	GLS	Tongue squamous cell carcinoma	↑	[76]
CircCOL1A1/miR-214-3p	GLS1	Colorectal cancer	↑	[73]
Circ_0001093/miR-579-3p	GLS1	Esophageal squamous cell carcinoma	↑	[71]
Circ_0075829/miR-326	GOT1	Pancreatic cancer	↑	[77]
Circ-MBOAT2/miR-433-3p	GOT1	Pancreatic cancer	↑	[78]
CircAKT3/miR-515-5p	SLC1A5	Gastric cancer	↑	[79]
Circ_0000069/miR-125a-5p	SLC1A5	Renal cell carcinoma	↑	[80]
Circ_0025033/hsa_miR-370-3p	SLC1A5	Ovarian cancer	↑	[81]
Circ-AGFG1/miR-497-5p	SLC1A5	Esophageal squamous cell carcinoma	↑	[82]
Circ_0000808/miR-1827	SLC1A5	Non-small cell lung cancer	↑	[83]
Circ_0000518/miR-330-3p	SLC1A5	Non-small cell lung cancer	↑	[84]
Circ_0001273/miR-622	SLC1A5	Esophageal cancer cells	↑	[85]
Circ-OXCT1/miR-516b-5p	SLC1A5	Non-small cell lung cancer	↑	[86]
Circ_0061558/miR-876-3p	SLC1A5	Triple-negative breast cancer	↑	[87]
Circ_0000463/miR-924	SLC1A5	Non-small cell lung cancer	↑	[88]
Circ-LDLRAD3/miR-137	SLC1A5	Non-small cell lung cancer	↑	[89]
Circ_0003602/MiR-149-5p	SLC38A1	Colorectal cancer	↑	[90]
CircRUNX1/miR-485-5p	SLC38A1	Colorectal cancer	↑	[91]
Hsa_Circ_0018189/miR-656-3p	SLC7A11, xCT	Non-small cell lung cancer	↑	[92]
Circ_0067717/miR-497-5p	SLC7A5	Colorectal cancer	↑	[93]
CircCPA4/miR-145-5p	ASCT2	Non-small cell lung cancer cells	↑	[94]
CircMAT2B/miR-491-5p	ASCT2	Head and neck squamous cell carcinoma	↑	[95]

Note: the symbol “(↓)” is used to indicate a decrease or inhibition of invasive transfer, whereas the symbol “(↑)” is used to indicate an increase or promotion of invasive transfer (the role of CirRNA)

## Glutamine metabolism and the tumour microenvironment

### Glutamine metabolism and immunity

#### Glutamine metabolism and T lymphocytes

Tumour immune escape is a phenomenon in which neoplastic cells evade recognition and elimination by the immune system through a variety of mechanisms, thus enabling their survival and proliferation within the organism. It was found that m6A modification-associated protein (IGF2BP3), which is highly expressed in human cervical cancer, can enhance Gln metabolism, promote lactate production and secretion, and affect Treg cell differentiation by up-regulating GLS and GLUD1, thus leading to immune escape [96]. Research has demonstrated a negative correlation between CD8 and the expression level of ASCT2. In the context of glutamine depletion or inhibition of glutamine transport, in conjunction with ICD-inducing chemotherapeutic agents, a synergistic activation of IFN-β occurs, leading to CD8 T-cell recruitment and an inhibition of cancer cell growth via the OTUD4/LDHA axis [97]. Additionally, there are critical interactions between metabolic byproducts and immune regulation. It has been demonstrated that enhanced glutamine metabolism results in the accumulation of toxic ammonia levels, which in turn damages lysosomes and mitochondria, leading to T-cell depletion. Consequently, strategies aimed at inhibiting ammonia transport have been proposed as a means of prolonging T-cell lifespans and enhancing antitumor efficacy [98]. Furthermore, exosome-derived circTRPS1 from bladder cancer (BCa) cells could regulate intracellular ROS homeostasis and promote CD8 T cell depletion via the circTRPS1/miR141-3p/GLS1 axis [99]. In contrast to the aforementioned findings, it was found that depletion of Gln or inhibition of glutamine metabolism in the tumor microenvironment leads to impaired T-cell function, which promotes immune escape of tumor cells and enhances cell proliferation, invasion, and metastasis. HCC cells have been observed to induce CTL (cytotoxic T lymphocyte) dysfunction via the Gln-GLS2-ERS pathway. In the absence of Gln in the medium, the levels of granzyme B (GZMB) and perforin (PRF1) secreted by CTLs co-cultured with HCC cells were reduced, and CTL function was decreased. It is evident that glutamine deprivation impairs the function of infiltrating CD8 + T cells in HCC and promotes tumour development [100]. This mechanism has been demonstrated in multiple myeloma, wherein silencing of SLC38A2 has been shown to result in reduced Gln uptake and T cell immune dysfunction [101]. Consequently, further exploration is necessary to elucidate the molecular correlation between the dynamic changes of glutamine metabolism, a pivotal metabolic pathway within the tumor microenvironment, and the extent of T-lymphocyte activation. Additionally, the mechanism by which these two factors synergistically regulate the proliferation of tumor cells warrants further investigation. In the future, it is necessary to systematically analyze the interaction between glutamine metabolism, T-cell immune function, and tumor cell proliferation through multi-omics integration analysis, dynamic metabolic flow tracking, and other technological means. This will provide an important theoretical basis for targeted metabolic interventions to enhance anti-tumor immune response.

A comprehensive investigation into the regulatory mechanisms of Gln within the immune microenvironment has led to the emergence of clinical validation for immune checkpoint blockade (ICB) therapy. The advent of programmed death-ligand 1/programmed death-1 (PD-L1/PD-1) checkpoint blockade immunotherapy represents a paradigm shift in the management of tumours. Nevertheless, the bulk of patients do not demonstrate a positive response to immunotherapy. The relationship between metabolic reprogramming in tumours, immune cells, and TME exerts a significant influence on tumour immune escape. The use of inhibitors of glutamine metabolism alone, while enhancing antitumor immunity, has some limitations. A recent study has demonstrated that 6-diazo-5-oxo-l-norleucine (DON) facilitates metabolic adaptations in tumor CD8 T cells, exhibiting promising clinical efficacy in ongoing trials [102]. In addition, JHU083, an inhibitor of glutamine metabolism, has been observed to increase CD8 T cell and CD4 Th1 cell infiltration, thereby enhancing the efficacy of PD-1 blockers. However, it is important to note that prolonged use of JHU083, while inhibiting glutamine metabolism, has been shown to lead to the upregulation of accumulated reactive oxygen species. This, in turn, results in the upregulation of PD-L1 in bladder cancer cells, the activation of the EGFR/ERK/C-Jun signaling pathway, and the facilitation of tumor immune escape [103, 104]. In this regard, a therapeutic strategy has been proposed that involves targeting glutamine metabolism in combination with PD-1/PD-L1 immune checkpoint blockade. This strategy shows promise as an anti-tumor immunotherapy. The present study will investigate the reversal of long-term glutamine blockade-induced upregulation of PD-L1 in bladder cancer cells through combination therapy with JHU083 and gefitinib [105]. In the context of studies examining lung and breast cancers, it has been observed that the upregulation of PD-L1 expression in cancer cells facilitates immune evasion. However, a concomitant decrease in glutamine uptake within cancer cells has been demonstrated to meet the demand for glutamine by lymphocytes within the tumor mesenchyme. This phenomenon subsequently results in a reduction in PD-L1 expression levels [106, 107]. In a study of triple-negative breast cancer (TNBC), it was found that when combined with the SLC7A5 blocker JPH203 and an anti-programmed cell death 1 (PD-1) antibody, it significantly inhibited cell proliferation, invasion, and migration, increased infiltration of CD8 + T cells, and suppressed tumor immune escape [106, 107]. A similar approach was employed in the context of TNBC, wherein molybdenum disulfide (MoS2) was utilized as a delivery vehicle for anti-PDL1 antibody (aPDL1) and V9302, to potentiate the anti-tumor immune response in TNBC cells and markedly impede TNBC tumor growth. Concurrently, an analysis predicated on two markers of T-cell activation, CD69 and CD25, revealed a substantial augmentation in the proportion of activated CD8 T cells within the tumor, along with a notable escalation in the concentration of cytokines in the interstitial fluid of the tumor. This analysis also demonstrated the merits of a minimal systemic toxic response and a paucity of therapeutic side effects [107, 108]. Specifically, V9302 has been shown to promote cancer cell autophagy, further enhance anti-tumor immunity by regulating ROS accumulation, decrease the expression of the T-cell co-suppressor molecule B7H3, and promote the production of GZMB by CD8 T cells [109]. Zhao L. et al. investigated the potential of a photodynamic immunostimulant consisting of a photosensitizer (dihydroporphyrin e6), an ASCT2 inhibitor (V9302), and a PD1/PDL1 blocker (BMS-1) to inhibit glutamine transport and GSH synthesis by targeting glutamine metabolism. The study found that this treatment could enhance the immune recognition function of CD8 + T cells and reduce the immune escape of tumor cells [110]. High GLS expression in colorectal cancer has been demonstrated to result in reduced T-cell infiltration and toxicity, which is a significant factor contributing to the poor prognosis observed in patients with this condition. Inhibiting GLS downstream of Glu-GSH fluxes activates ROS-related signaling pathways in tumor cells, thereby promoting immunoproteasome activity to enhance tumor immunogenicity. This is a major reason for the poor prognosis of colorectal cancer when compared to any single therapy. The combination of Glu-GSH flux inhibitors and anti-PD-1 antibody combination therapy demonstrated superior tumor growth inhibition compared to any single therapy [111]. Hypoxia-induced GCN5-mediated acetylation of GLS2 at the K151 site has been shown to enhance the interaction of GLS2 with YAP1. This, in turn, has been demonstrated to result in the up-regulation of PD-L1 expression, the reduction of CD4 and CD8 T-cell activation and tumor infiltration, and the promotion of the growth of pancreatic ductal adenocarcinoma (PDAC) cells [112]. Therefore, when the GCN5 inhibitor MB-3 is administered upstream of glutamine metabolism, the combination of anti-PD-1 antibodies and immune checkpoint blockade results in the elimination of tumor immune evasion and the enhancement of antitumor efficacy. Pyrroline-5-carboxylic acid reductase 1 (PYCR1) has been identified as a key player in the progression of lung cancer, functioning through a metabolic linkage between proline and glutamine. Upregulated PYCR1 expression has been demonstrated to activate STAT3 phosphorylation in lung cancer, which in turn leads to elevated PD-L1 expression and subsequent inhibition of T cell infiltration [113].

Chimeric antigen receptor-T (CAR-T) cell therapy has demonstrated remarkable success in eradicating hematologic malignancies; however, its efficacy in treating solid tumors has been limited by the presence of immunosuppressive TME. To this end, the researchers developed genetically programmable nanoparticles (D@aPD-L1 NVs) loaded with glutamine antagonists, which deliver glutamine antagonists targeted to the tumor site. This effectively reduces the number of immunosuppressive cells and enhances the anti-tumor ability and long-term memory immunity of CAR-T cells. It is hypothesized that these nanoparticles will enhance the therapeutic effect of CAR-T cells on solid tumors [114]. CAR-T cells suffer from the disadvantage of insufficient persistence in vivo. In contrast, TNor TCMs have greater renewal capacity and anti-tumor immunity compared to TEMor Teff. To address this issue, the researchers added the glutamine antagonist DON to the cultures. The results demonstrated that CAR-T, which has enhanced mitochondrial OXPHOS that utilizes fatty acids and reduced glycolytic activity, demonstrated more robust tumor elimination [115]. A prospective approach to enhance the efficacy of CAR-T cell therapy involves the modulation of metabolic and differentiation status through the ex vivo inhibition of glutamine.

#### Glutamine metabolism and tumour-associated macrophages (TAMs)

Tumor-associated macrophages (TAMs), which constitute an important part of the tumor microenvironment (TME), are classified into M1-type macrophages (M1-TAMs) and M2-type macrophages (M2-TAMs). M1-TAMs have been shown to inhibit tumor growth, while M2-TAMs have been shown to promote tumor growth [116]. TAMs have been shown to play an important role in tumor cell genesis, development, and metastasis by regulating multiple signaling pathways. Research has demonstrated that IL-6, secreted by M2-TAMs, can induce glutamine transaminase 2 (TGM2) expression through the activation of the JAK/STAT3 signaling pathway [117]. This, in turn, has been shown to further enhance the EMT and invasive ability of breast cancer cells. Moreover, it has been demonstrated that the over-expression of TGM2 enhances IL-1β-induced macrophage recruitment and chemokine secretion, whilst concomitantly promoting the activation of the NF-κB signalling pathway [118]. The TFAP2A/NEL3 axis has also been found to promote M2 polarisation through Gln metabolism, thus providing a theoretical basis for immunotherapy targeting cancer TMEs [119].

It has been demonstrated that folic acid-targeted nanoparticles (FA-DCNP) loaded with glutamine inhibitor DON and calcium carbonate CaCO3 can target M2-TAMs and attenuate M2-TAMs activity. This, in turn, has increased the proportion of M1-TAMs and effective inhibition of cancer cell proliferation. Consequently, this has led to an improvement in the tumor immune microenvironment and enhancement of anti-tumor immunity [120]. Concurrently, the release of DON and calcium carbonate produces synergistic antitumor effects by hindering glutamine metabolism and inducing calcium overload. Concurrently, DRP-104, the prodrug of DON, has been observed to promote the polarization of tumor-associated macrophages (TAMs) to the M1-TAMs phenotype. This process has been demonstrated to play a pivotal role in enhancing anti-tumor immunity and inhibiting tumor proliferation [121]. Photodynamic therapy also plays an important role in the polarization of TAM. C9SN, a carrier-free immunotherapeutic nano-enhancer with dual synergistic effects, was constructed using self-assembly of the GLS inhibitor compound 968 (C968) and the photosensitizer Chlorin e6. C9SN modifies the immunosuppressive tumor microenvironment (ITM) by impeding glutamine metabolism on one hand, polarizing M2-TAMs to M1-TAMs, which in turn recruits and activates CTLs, while on the other hand, C968 in the nanoaugmentation amplifies the intracellular oxidative stress by inhibiting glutamine metabolism-mediated GSH deprivation, which results in severe cell death, and also enhances the immunogenic cell death (ICD) effect [122]. The inhibitor of glutamine synthetase (GS) activity, l-methionine sulfoximine (MSO), has been shown to inhibit glutamine metabolism, induce a shift to M1-TAMs, and act as a potent antitumor immunity [123]. A recent study has indicated that SLC7A5, SLC7A8, SLC38A1, and SLC38A2 may play a regulatory role in TAM polarization. This finding emerged from a comprehensive bioinformatics analysis that evaluated the SLC-associated glutamine transporter proteins in the context of breast cancer patient prognosis. However, it is crucial to note that further in-depth and systematic investigation is necessary to elucidate the specific mechanisms underpinning these observations [124].

CD47 is an inhibitory receptor that is expressed on the surface of normal cells and tumour cells. It is involved in a variety of physiological processes, including cell proliferation, apoptosis, migration and immunity. In the immune response, tumor cells exhibit high levels of CD47 expression, which impedes phagocytosis by macrophages through its binding to SIRPα on the macrophage surface, thereby facilitating immune evasion. Research has shown that chemotherapy can trigger immune evasion of tumor cells, which is a significant contributing factor to patients’ vulnerability to relapse and high mortality. Following chemotherapy, macrophages secrete interleukin-18 (IL-18), which in turn upregulates L-amino acid transporter protein 2 (LAT2) expression in tumour cells. This has been shown to significantly enhance the uptake of leucine and Gln, and to further activate the mTORC1 signalling pathway, thus promoting immune escape [125]. Consequently, enhancing the sensitivity of chemotherapy by inhibiting the uptake of LAT2-mediated amino acids to reduce CD47 and by enhancing the infiltration and phagocytosis of tumour cells by macrophages has emerged as a potential strategy for the treatment of cancer.

#### Glutamine metabolism and NK cells

Gln has been identified as a significant component that exerts a considerable influence on the proliferation and functionality of natural killer (NK) cells. Research has shown there is a dependence of NK cells on Gln, and an elevation in NK cell proliferation is observed with increasing Gln concentrations [126]. The reason for this is that, under steady state, the level of Gln in natural killer T (NKT) cells is higher than that in CD4 + T cells, and when activated, NKT cells increase the breakdown of Gln. Activated NKT cells use Gln to provide fuel for the TCA cycle and GLULH synthesis, thereby increasing the proliferation capacity of NK cells. Consequently, it can be deduced that each branch of Gln metabolism appears to be critical for NKT cellular homeostasis and mitochondrial function. In contrast, studies have demonstrated that the inhibition of glutamine metabolism in cancer cells using the DON precursor drug DRP-104 results in an augmentation of the number of NK and NK T-cells within the immune microenvironment, thereby enhancing anti-tumor immunity [121]. It has also been demonstrated that the long non-coding RNA PWAR6, which is overexpressed in metastatic colorectal cancer tissues, functions as a competitive inhibitor of Keap1, thereby promoting the stabilization of NRF2. This, in turn, results in the upregulation of SLC38A2 expression, the enhancement of glutamine uptake and depletion of glutamine from natural killer (NK) cells, and the promotion of immune escape [127]. Consequently, there is a necessity to investigate the effect of glutamine metabolism and NK cell interaction on the proliferative effects of cancer cells in a more in-depth and broader systematic manner through multidimensional modeling and the integrated use of multiple biological approaches.

### Glutamine metabolism and cancer-associated fibroblasts(CAFs)

CAFs represent a pivotal element of the TME, exerting a pivotal function in shaping the TME. However, research into this field remains in its infancy. Despite the rapid advances witnessed in metabolic therapies over the past decade, their efficacy against a multitude of tumours has remained inconsequential. The rationale behind this phenomenon pertains to the function of CAFs in regulating ECM remodelling within the TME. The TME, in turn, interacts with cancer cells, thereby promoting their growth [128]. Consequently, the targeting of CAFs in conjunction with glutamine metabolism within the context of cancer treatment has emerged as a promising therapeutic strategy [129].

In their seminal study, He et al. discovered that oestrogen-activated GPER in CAFs promotes the expression of GLUL and lactate dehydrogenase B (LDHB), enhances the survival of Triple Negative Breast Cancer(TNBC) cells both in vitro and ex vivo, and increases chemotherapy resistance [130]. The present study has demonstrated that CAFs increase the proliferation of hormone-sensitive human prostate cancer cells (LNCaP) and lung adenocarcinoma (LUAD) cells, and promote glutamine metabolism [131, 132]. CAFs have been shown to play a pivotal role in mediating interactions between cancer cells and TME via exosome transfer. As demonstrated in the extant literature, METTL3 in CAFs-derived exosomes has been shown to promote proliferation, invasion, and glutamine metabolism in non-small cell lung cancer (NSCLC) cells by inducing m6A modification of SLC7A5 and stabilising its expression [133]. In addition, the exosome of CAFs, LINC01614, has been demonstrated to directly interact with ANXA2 and p65, thereby promoting NF-κB activation. This, in turn, results in the upregulation of glutamine transporter proteins, SLC38A2 and SLC7A5 [132]. Consequently, this process leads to enhanced Gln influx into cancer cells and is associated with a poor prognosis. Evidence suggests that the targeting of specific exosome subtypes in CAFs to inhibit Gln uptake and tumour progression has therapeutic potential.

As CAF-targeted therapies are progressively attracting attention, a significant number of studies are investigating the use of CAF as a potential breakthrough to overcome the limitations of tumour therapy. Modifying the TME is a complex process. Research has demonstrated that augmented glutamine metabolism in CAFs gives rise to collagen-rich extracellular matrix and fosters tumor development [134, 135]. Concurrently, Netrin G1 (NetG1) was identified as a pivotal initiator of PDAC tumorigenesis. Furthermore, CAFs support PDAC survival through NetG1-mediated enhancement of Gln metabolism [136]. Consequently, the combination of NetG1 blockade and glutamine metabolism inhibition utilizing a neutralizing antibody could impede the development of PDAC tumors. Ai C et al. developed a controlled-release nanodroplet targeting CAFs by co-delivering the ASCT2 (SLC1A5) inhibitor V9302 and GLULsiRNA (siGLUL) to CAFs. This approach resulted in the disruption of glutamine metabolism interactions between CAFs and cancer cells, the blocking of activated CAFs, and a reduction in extracellular matrix production [137]. Evidence suggests that the targeting of CAFs could have significant applications.

## Multidimensional investigation of potential anti-cancer ‘targets’ of glutamine metabolism and their inhibitors

### Targeting glutamine metabolism-related enzymes and their inhibitors

As indicated by the aforementioned evidence, it can be hypothesised that ‘glutamine-addicted’ cancer cells are reliant on GLS for survival. Furthermore, GLS has been identified as the first rate-limiting enzyme in glutamine metabolism, which has emerged as a potential therapeutic target for tumours.

To investigate the role of GLS in cancer, we studied several GLS inhibitors. Common examples include CB-839, JHU-083, DON (6-diazido-5-oxoleucine), BPTES (bis-2-(5-phenylacetamido-1,3,4-thiadiazol-2-yl)ethylsulfide)and968 (5-(3-bromo-4-(dimethylamino)phenyl)-2,2-dimethyl-2,3,5,6-tetrahydrobenzo[a]phenanthren-4(1 H)-one), Hexylselen(CPD-3B), Ebselen, etc.(Table 2). The present inhibitor, CB-839, has been demonstrated to engender anticancer effects in a variety of cancers, including myeloproliferative neoplasms, CRC [138, 139]. It was found that CB-839 could synergize with other anticancer drugs to exert beneficial therapeutic effects, and KRAS mutation led to enhanced glucose and glutamine metabolism in ovarian cancer cells, which was better inhibited by the combination of metformin and CB-839 [32]. The combination of CB-839 and azacytidine (AZA) was found to be synergistic in a single-arm, open-label, phase 1b/2 study conducted in patients diagnosed with advanced myelodysplastic syndromes (MDS). This study comprised a dose-escalation phase, which involved six participants, and a dose-expansion phase, which included twenty-four participants. The treatment was well tolerated, with an objective remission rate of 70% and complete remission in 53% of participants (bone marrow). These data demonstrate the safety and efficacy of CB-839 and AZA as a combined metabolic and epigenetic approach to treating MDS [140]. However, CB-839 exhibits differential therapeutic effects on various tumors, and glutamine catabolism contributes to the viability of refractory multiple myeloma (MM) cells. Moreover, CB-839 has been demonstrated to inhibit myeloma cell proliferation and enhance sensitivity to histone deacetylase (HDAC) inhibitors, suggesting that CB-839 has potential as a therapeutic agent for patients with MM [141]. However, GLS inhibition was found to impair CD8 + T-cell activation in STK11-/LKB1-deficient lung cancer, and the use of CB-839 to inhibit CD8 + T-cell expansion negatively impacted tumor therapy [142]. Consequently, further systematic studies are required to elucidate the synergistic mechanism and the optimal combination of CB-839 with other drugs or therapeutics. The inhibitor DON has been shown to irreversibly inhibit GLS activity. To enhance the efficiency of the process, the precursor ‘JHU-083’ was synthesised. This has been demonstrated to inhibit the proliferation and metastasis of thyroid cancer and to enhance the innate immune response [143]. Concurrently, JHU-083 demonstrated remarkable efficacy in impeding the proliferation of EGFR-driven lung tumors, fostering adaptive T cell-mediated tumor-specific immune responses [103]. Inhibitor 968 has been demonstrated to induce cell cycle arrest in G1 phase and to increase cellular ROS production, promote cellular stress and induce apoptosis in cancer cells, and inhibit the AKT/mTOR/S6 signalling pathway. These finding suggest that 968 may represent a promising therapeutic approach for the treatment of human endometrial cancer [144]. It was also found that 968 was capable of reversing adriamycin resistance in MCF-7/ADR, a drug-resistant cell line of BC, in a time- and drug-concentration-dependent manner [145]. CPD-3B@SOL micelles demonstrated adequate metabolic stability in both blood and liver microsomes. These advantages significantly enhanced the bioavailability and antitumor efficacy of CPD-3B@SOL micelles in an in vivo H22 hepatocellular carcinoma xenograft mouse model [146]. Interestingly, the inhibitor CPD-3B is virtually non-toxic to normal cells, while targeting not only GLS1 and GDH but also thioredoxin reductase (TrxR) and amidotransferase (GatCAB), resulting in the selective elimination of cancerous cells through a multifaceted mechanism. This three-pronged approach has demonstrated significant anticancer efficacy in xenograft models [147]. However, it should be noted that no human experimentation has been conducted thus far. The combination of gene-targeted interventions and glutamine metabolism blockade has been shown to exhibit superior therapeutic efficacy in tumor suppression compared to monotherapy. HUR has been identified as a regulator of GLS mRNA alternative splicing and isoform translation/stability in breast cancer. A therapeutic strategy that involves dual inhibition of GLS and HUR has been proposed as a treatment for breast cancer [148]. The combination of glutamine metabolism inhibition and lysosomal inhibition demonstrates therapeutic potential in the treatment of glioblastoma (GBM) [24]. The combination of 968 and PD-L1 has been shown to enhance the body’s immune response to ovarian cancer, and the combination of 968 with the autophagy inhibitor chloroquine (CQ) has been demonstrated to have a synergistic effect on the growth of NSCLC cells [149, 150]. GLS enhances glycolysis in esophageal squamous cell carcinoma (ESCC) by interacting with PDK1, so co-targeting GLS and PDK1 may be a novel therapeutic approach for ESCC patients [151]. Furthermore, several novel GLS inhibitors have exhibited superior therapeutic efficacy in clinical settings and preliminary studies, such as IN-3, have been shown to possess antiproliferative effects on PCa. The selective inhibitor compound 27 (IPN60090), which is currently in a first-in-class clinical trial, focuses on optimising physicochemical and pharmacokinetic properties, with a strong in vivo target engagement capability, and should strongly inhibit GLS in humans [152, 153]. BPTES-loaded bionic Cu-doped polypyrrole nanoparticle (CUP) nanosystems (PCBs) effectively inhibited GLS1 activity, thereby decreasing GSH content and consequently suppressing primary and metastatic tumors. This represents the first instance of a GLS inhibitor being applied to enhance tumor cell copper death and immunotherapy [154]. GLS represents a pivotal target in clinical applications, particularly in the context of anti-cancer therapy and other therapeutic areas. However, the majority of GLS inhibitors exhibit poor solubility, inadequate selectivity, and limited bioavailability, among other inherent limitations. Consequently, the majority of these inhibitors have yet to meet the stringent criteria necessary for clinical utilisation, predominantly remaining at the preclinical research stage.

**Table 2:** Mechanistic role of various glutaminase inhibitors

Inhibitors	Cancer Type	Cell Line/Experiment Type	Role	Refs
CB-839	Triple-negative breast cancer	HCC1806 T47D	A marked decrease in glutamine consumption, glutamate production, and oxygen consumption	[155]
DON	Pancreatic ductal adenocarcinoma	HPAF-II, BXPC-3 etc. C57BL/6J NU/J Anymic	Reduce asparagine production by inhibiting asparagine synthetase (ASNS)	[156]
BPTES	Glioma	Human IDH1	Selectively slow growth in cells with IDH1 mutations	[157]
968	Endometrial cancer	Ishikawa HEC-1B	Induce cell cycle arrest in the G1 phase and increase cellular ROS production	[144]
JHU-083	Glioma	IDH1R132H	Disrupt mTOR signaling and downregulate CyclinD1 protein expression	[158]
CPD-3B	Colon cancer	HL7702 HCT116 etc.	Target not only KGA and GDH but also thioredoxin reductase (TrxR) and amidotransferase (GatCAB)	[147]
Ebselen			Target proteins through redox reactions with selenocysteine/cysteine residues	[159]

It is also of interest to note the requirement of an essential enzyme, GDH, after the conversion of glutamine to glutamate. A significant body of research has identified a correlation between elevated GDH levels and a wide spectrum of diseases, with some studies even suggesting its use as a prognostic marker for colorectal cancer metastasis [160]. Consequently, GDH emerges as a promising therapeutic target during tumour growth, proliferation, invasion, and metastasis. Inhibition or down-regulation of GDH expression leads to a reduction in α-KG production, which in turn affects the normal physiological activity of the tumour in a manner that reduces energy supply.

In the course of the present study, the following inhibitors of glutamate dehydrogenase were identified: epigallocatechin-3-gallate (EGCG), hexachlorophene (HCP), bithionol (BTH), GW5074, CPD-3B, ebselenin, R162, and others(Table 3). In particular, EGCG has been shown to impede the progression of liver fibrosis by inhibiting GDH enzyme activity and glutamine metabolism, thereby further reducing the risk of hepatocellular carcinoma [161]. The present study proposes a novel combination therapy for breast cancer that involves the utilization of EGCG-enabled phosphatase and acidic dual-responsive nanotherapeutic agents, which demonstrate the capacity to penetrate deep tumor tissue. Recent studies have demonstrated the feasibility of synthesizing a novel metal-polyphenol-based multifunctional nanomedicine (Fe-dbef) containing various inhibitors, such as EGCG and BPTES, among others. These inhibitors have been shown to exhibit highly efficient antiproliferative properties in pancreatic cancer [162]. In comparison with GLS inhibitors, a paucity of research has been conducted on GDH inhibitors, and there are no drug currently available for clinical application. As one of the most significant anticancer targets in glutamine metabolism, there is considerable potential for future research and development.

**Table 3:** Mechanistic role of various GDH inhibitors

Inhibitors	Cancer Type	Cell Line/Experiment Type	Role	Refs
EGCG	Triple-negative breast cancer	TNBC tumor-bearing mice	GLULH peroxidase by regulating mitochondrial glutamine metabolism	[163]
ECG	Prostate cancer	PC3,22RV1	Impairs the synthesis of fatty acids via inhibition of PI3K/AKT/mTOR signaling pathway	[164]
HCP			Form a ring around the internal cavity in GDH through aromatic stacking interactions	[165]
BTH	Acute myeloid leukaemia	NSG mice	Increased mitochondrial superoxide levels	[166]
GW5074			Bind as pairs of stacked compounds at hexameric 2-fold axes	[165]
CPD-3B	Colon cancer	HL7702 HCT116 etc.	Target not only KGA and GDH but also thioredoxin reductase (TrxR) and amidotransferase (GatCAB)	[147]
Ebselen			Target proteins through redox reactions with selenocysteine/cysteine residues	[159]
R162	Non-small cell lung cancer	E.coli DH5α strain E. coli BL-21 strain	Overcome both acquired resistance and EMT-induced metastasis in vivo	[167]

### Targeting glutamine metabolism transporter inhibitors

Gln metabolism has received increasing attention in the process of material transport and energy metabolism in cancer cells, and increased Gln catabolism in cancer cells has been associated with increased expression of Gln transporter proteins. Mediating this property, it is clinically possible to inhibit the expression of Gln transporter proteins, making it one of the effective methods for cancer treatment. Among them, ASCT2 is considered to be the major Gln transport protein in cancer cells.

ASCT2 is primarily responsible for the transmembrane transport of Gln and some macromolecular neutral amino acids. These amino acids are indispensable for cell survival, metabolism, signal transduction, and processes such as autophagy. To date, ASCT2 is an important target in cancer development. The design of drug targets for ASCT2 typically follows a substrate analogue approach, wherein substrate analogues function as competitive inhibitors, thereby reducing the uptake of essential amino acids by cancer cells. The existence of several amino acid analogues that can compete with ASCT2 substrates for binding sites and reduce amino acid translocation has been reported(Table 4) [168]. A clinical study demonstrated that GPNA suppressed the expression of ASCT2 and diminished the uptake of Gln by cancer cells [169]. A study indicated that the simultaneous use of GPNA to block ASCT2, to inhibit the uptake of Gln by cancer cells, significantly enhanced the inhibitory effect of cetuximab on the proliferation of gastric cancer cells [170]. Furthermore, the combination of cetuximab and GPNA in gastric cancer cells significantly induced apoptosis and exhibited a more potent inhibitory effect on gastric cancer proliferation in vitro and in vivo than either treatment alone. Several studies have been conducted by scholars on the potential of GPNA to inhibit the non-sodium transport system of neutral amino acids. These studies have revealed that GPNA exerts an inhibitory effect on Gln uptake in both LAT1- and LAT2-expressing cancer cell lines [171]. This finding indicates that GPNA can significantly impede the uptake of Gln by cancer cells, thereby reducing intracellular Gln levels. In addition, some scholars have investigated the high affinity of ASCT2 and ASCT1 for binding to amino acid substrates. They found that the apparent affinity of the substrate in ASCT1 is 2–5 times higher than that in ASCT2 in HEK-293 cells. However, the study showed that glutamine binds only to ASCT2 [172]. A subsequent study demonstrated that the binding affinity of GPNA for ASCT2 surpassed that of ASCT1 [173]. Therefore, GPNA has been shown to inhibit the uptake of glutamine by cancer cells by suppressing the expression of ASCT2, exhibiting a powerful effect, and also in this study, Benzylserine and Benzylcysteine were found to competitively inhibit the substrate-binding site of ASCT2 and reduce the uptake of Gln. However, due to the low binding affinity of Benzylserine and Benzylcysteine to ASCT2, high concentrations of inhibitors are required to compete for the substrate binding site. This may lead to enhanced binding of inhibitors to other proteins. Consequently, researchers have developed a competitive antagonist with a high affinity for ASCT2, designated V-9302 [174]. To confirm the hypothesis that V-9302 and ASCT2 can bind to each other, the researchers used HEK-293 cells expressing a tetracycline-inducible ASCT2 vector. Using the drug affinity response target stability technique, it was possible to observe that ASCT2 was protected from protein hydrolysis in a V-9302 concentration-dependent manner. It can thus be hypothesised that there is a stable interaction between V-9302 and ASCT2. Furthermore, it was determined that ASCT1 demonstrated instability in the presence of V-9302, suggesting that V-9302 exhibited a high degree of affinity for ASCT2. The V-9302 inhibitor is subject to certain limitations. Firstly, it can be displaced from the binding pocket by endogenous substrates, which consequently hinders its efficacy. In contrast, GPNA is a non-specific inhibitor of ASCT2. It has also been demonstrated to impair the uptake of essential amino acids by the transporter protein SLC38A2, thereby decreasing the cellular levels of these nutrients [175]. Consequently, there is a necessity to design compounds capable of irreversibly targeting and blocking ASCT2 transporter proteins.

In recent years, with the increasing resolution of the ASCT2 3D structure, cysteine residue C467 is an important part of the composition of the ASCT2 substrate binding site, and residue C467 plays a key role in recognizing and binding glutamine [176]. Therefore, the inhibitors developed for the C467 residue of ASCT2 boast the advantages of high specificity and high potency, which can effectively avoid the loss of competitive inhibition by substrate analog inhibitors due to high substrate concentration. The design of inhibitors targeting C467 has created new opportunities for the field of drug discovery and development. Through rational medicinal chemistry approaches, the development of inhibitors with high affinity and selectivity against ASCT2 is possible. For instance, the presence of small molecules containing acrylamide has been observed to result in the formation of covalent adducts with cysteine through a process known as Michael addition reaction, thereby achieving irreversible inhibition [177]. This particular targeted therapy is currently in the preliminary stage of drug screening, and its potential to provide new therapeutic modalities for various types of cancers is under active investigation. In the future, in-depth analysis of the ASCT2 3D structure using structural biology techniques and an in-depth understanding of the interaction mechanism between ASCT2 and inhibitors will help to optimize the structure of inhibitors and improve their efficacy.

**Table 4:** Pharmacological inhibitors of glutamine transporter proteins

Inhibitors	Cancer Type	Cell Line/Experiment Type	Role	Refs
GPNA	Breast cancer	MCF-7	Inhibition of Na^+^-dependent vectors	[178]
Benzylserine	Breast cancer	MCF-7	Substrate binding site for competitive binding of ASCT2	[179]
Benzylcysteine	Normal kidneys	HEK293	Binding of substrates that prevent transport to ASCT2	[180]
V-9302	Clear-cell renal-cell carcinoma	UM-RC-3	Inhibition of ASCT2-mediated glutamine uptake	[181]
Phenylglycine analogues	Normal kidneys	HEK293	Binds to ASCT2 cysteine residues	[182]
γ-FBP	Melanoma	C8161	Generate a large outward current	[183]

### Derivatives of clinical novel inhibitors

Despite the advances made in GLS inhibitors, the development of precursor drugs has encountered significant setbacks due to a lack of selectivity and low bioavailability. This has resulted in some cancer cells exhibiting resistance to GLS inhibitors. Consequently, the quest for effective and highly selective GLS inhibitors remains an open question. Docking simulations based on the complex crystal structures of GLS1 and its inhibitor CB-839 revealed that bearing thiadiazole skeletons exhibit GLS1 inhibition. Furthermore, it was determined that 27 thiadiazole derivatives had been synthesised, with 4d exhibiting a greater degree of GLS1 inhibitory activity than the known GLS1 inhibitors, DON and A. 4d is a highly promising novel inhibitor of GLS1 [184]. Methane dibenzo[b, f] [1, 5]dioxin, a novel dioxin derivative, has been identified as a novel GLS inhibitor with potential anti-glioblastoma (GBM) properties. The mechanism of action of this compound involves the promotion of apoptosis by increasing the production of ROS in two types of GBM cells. Additionally, it has been observed to exhibit anti-migratory and anti-proliferative properties over time [185]. However, it is important to note that other types of derivatives may be hepatotoxic. Recent studies have demonstrated the potential of glutamine-coupled organophilin (IV) compounds as chemotherapeutic agents for colon cancer. These compounds exhibit a high degree of DNA/protein affinity, favorable in silico ADME profiles, and pronounced antiproliferative activity. In experimental models of colon carcinogenesis induced by DMH/DSS, these compounds have been observed to attenuate tumor burden and volume, inhibit cell proliferation, and induce apoptosis, with minimal toxicity. These findings support the further investigation of glutamine-coupled organophilin (IV) compounds as potential chemotherapeutic agents for the treatment of colon cancer [186]. L-glutaminase purified from Klebsiella pneumoniae (AS KP23) has been shown to exhibit significant toxicity against human hepatocellular carcinoma (HEPG-2) and breast cancer cell lines [187].

The utilisation of combination drug holds considerable promise, as they can circumvent the limitations inherent in single-agent drug. Moreover, combination therapy has the potential to enhance the number of anticancer targets and exert a synergistic effect. Consequently, it is feasible to combine other cancer treatment modalities with glutamine metabolism inhibitors, a combination that will prove to be of significant benefit in the treatment of cancer.

Abnormal proliferation of cancer cells accompanied by exuberant nutrient metabolism is one of the main physiological characteristics of cancer, and starvation therapy has now become a current research hotspot in the field of oncology. Starvation therapy is a strategy to treat cancer by depriving tumours deprived of key nutrients [188]. In recent years, mitochondria have been identified as a critical component in the genesis and progression of tumour stem cells. Consequently, the development of new small molecules with the capacity to target mitochondria is anticipated to eradicate tumour stem cells. Lonidamine (LND) is a derivative of indazole-3-carboxylic acid. LND selectively inhibits aerobic glycolysis and energy metabolism in tumour cells [189–191]. A related study found that LND and BPTES were able to induce glycolysis and inhibit glutamine metabolism, which was accompanied by significant mitochondrial damage, ultimately blocking energy supply and posing a threat to tumour survival, to efficiently kill tumours. Nevertheless, the efficacy of starvation therapy facilitated by LND is constrained by the inefficiency of drug transportation, off-target effects, and compensatory glutamine metabolism [192]. In this regard, the finding of a recent study have indicated that a novel LND derivative, HYL001, can selectively target mitochondria and effectively inhibit cancer stem cells (CSC)(Table 5) [193]. The HYL001 compound exerts its main mechanism of action through the down-regulation of GLS expression, thereby impeding glutamine metabolism. Consequently, HYL001 has demonstrated notable antitumour activity in vivo, whether as a monotherapy or in combination with another anticancer drug. However, it should be noted that the majority of current studies are still in the preclinical stage and have not yet been approved for entry into clinical therapy. Consequently, an exhaustive examination of mitochondrial function and energy regulation mechanisms will establish the theoretical foundation for targeting mitochondria in cancer treatment. Thus, the combination of inhibitors targeting mitochondria and glutamine metabolism is anticipated to emerge as a novel and promising strategy for cancer treatment.

Cancer remains one of the incurable diseases in the world. The main clinical treatments for cancer to date have been surgical resection, radiotherapy, and chemotherapy [194]. Early chemotherapy is chiefly focused on the direct targeting of the DNA strand, with platinum compounds, comedones, vincristine, paclitaxel, and anthracyclines being the most commonly used [195–199]. One of these agents, retinoic acid (RA), is a chemotherapeutic agent used to induce neuronal cell differentiation in neuroblastoma. While N-(4-hydroxyphenyl) retinamide (4-HPR), a derivative of RA, is a potent chemopreventive and antiproliferative agent against various cancers [200, 201]. A phase I-III clinical trial of the retinoic acid derivative FER in metastatic breast cancer (BC) was previously conducted. However, the trial was discontinued due to the drug’s limited therapeutic efficacy and poor bioavailability. Additionally, the clinical trial was terminated due to the occurrence of side effects, including impaired dark color adaptation [200, 202]. In order to reduce the side effects, p-dodecylaminophenol (p-DDAP) was further designed, and it has been demonstrated to be effective against a wide range of cancers [200]. As chemotherapeutic drug currently in clinical use are cytotoxic drug, they all damage normal cells to varying degrees, and various toxic side effects (e.g. nephrotoxicity, hepatotoxicity, etc.) occur. To date, there has been no treatment discovered that combines glutamine metabolism and chemotherapy, and a combination of the two may result in unintended consequences.

The advent of cancer immunology research has precipitated the emergence of immunotherapy as a leading treatment modality within the contemporary oncological domain. This novel approach boasts three significant advantages over conventional therapeutic modalities: namely, long-term efficacy, a reduced incidence of adverse effects, and the capacity to address tumour heterogeneity. Inhibitors of glutamine metabolism have been demonstrated to achieve inhibition of glutamine (Gln) metabolism with activation of the body’s immune defenses in tumor cells. In a series of studies, CB-839, a drug designed to target glutamine metabolism, has been shown to effectively block the IL-4-induced anti-inflammatory phenotype in macrophages. Additionally, CB-839 has been observed to enhance these cells’ tumor-killing capacity [203]. It has been shown that tumor cells and type 1 conventional dendritic cells (cDC1) compete for Gln uptake via the transporter protein SLC38A2 to modulate anti-tumor immunity [204]. Tumor cells and tumor-infiltrating myeloid cells have been observed to compete for glutamine uptake via the transporter protein SLC1A5. This competitive process plays a crucial role in regulating antitumor immunity. A study was conducted to determine the effects of glutamine uptake limitation in hepatocellular carcinoma cells and supplementation with IRE1α/XBP1 signaling or glutamine blockade on the immunosuppressive effects of GPR109A + myeloid cells and tumor progression. The results showed that these interventions effectively abrogated the immunosuppressive effects of GPR109A + myeloid cells and slowed down tumor progression. A recent study identified an immunometabolic crosstalk between hepatocellular carcinoma cells and myeloid cells that promotes tumor progression through the glutamine metabolism/ER stress/GPR109A axis. These findings suggest that GPR109A can be utilized as an immunometabolic checkpoint and propose an immunometabolic checkpoint for cancer therapy [205]. JHU-083 has been demonstrated to be highly effective against EGFR-driven lung tumorigenesis, with the capacity to promote adaptive T-cell-mediated tumor-specific immune responses that enhance evasion [103]. However, the efficacy of glutamine inhibitors in selectively targeting certain tumors while concurrently impeding the immune response of the host organism is a salient concern. It has been demonstrated that the deprivation of glutamine following the administration of inhibitors of glutamine metabolism confers immunotherapy resistance by inhibiting IFN-γ signaling in cancer cells [206]. Recent studies have demonstrated the potential of the Glutamine Metabolism Immunity Index (GMII) to serve as a reliable prognostic tool and to accurately predict the response to immunotherapy in bladder cancer patients, in addition to identifying candidate small-molecule drug. Furthermore, the novel ‘glutamine metabolism-related genes’ guidance strategies for predicting survival and chemo-immunotherapy efficacy may also apply to cancers other than bladder cancer [207]. Overcoming immunosuppression in the tumor microenvironment (TME) is critical for the development of novel cancer immunotherapies. IL-16 administration improved anti-tumor immune responses by enhancing Th1 cell polarization through the inhibition of glutaminase catabolism via the down-regulation of glutaminase in CD4 + cells [208]. Consequently, the targeting of immunotherapy and the modulation of the immune microenvironment using glutamine metabolism regulation could also be a potential target for cancer treatment. It is important to acknowledge that immunotherapy drug for cancer are still under constant research and development, and that further verification of their efficacy and safety is required through additional clinical trials and studies.

**Table 5:** Pharmacological effects of derivative inhibitors

Inhibitors	Cancer Type	Cell Line/Experiment Type	Role	Refs
Methanodibenzo[b, f][1,5]dioxocins	Glioblastoma	SNB19	Increases the production of reactive oxygen species	[185]
Lonidamine	Hepatocellular carcinoma	LM3	Damage to mitochondria	[190]
HYL001	Breast cancer	4T1	Down-regulated GLS expression	[193]
Inosine	Melanoma	Mice	Enhances T cell-mediated cytotoxicity	[209]
L-GLS	Breast cancer Hepatocellular carcinoma	Human hepatocellular and breast cancer cell lines	Reduce the survival rate of cancer cells in a dose-dependent manner	[187]

## Glutamine metabolism and drug resistance

Glutamine plays a pivotal role in the progression of tumors, and the strategic limitation of its availability is emerging as a potential therapeutic approach. As previously stated, despite the development of inhibitors targeting “targets” associated with the glutamine metabolic pathway, and significant advancements in therapeutic strategies such as surgical resection, radiotherapy, targeted therapy, and immunotherapy, the metabolic plasticity of tumor cells contributes to their adaptability to glutamine limitation. This is a primary factor contributing to the development of drug resistance. At present, drug resistance continues to be a significant contributing factor to the suboptimal patient prognosis. The development of drug resistance in cancerous cells is a major cause of concern, as it not only leads to tumor recurrence but also perpetuates the cancer, resulting in poor patient survival. Cancer cells manifest a variety of adaptations in response to metabolic challenges [210]. In conditions where glutamine metabolism is inhibited, these adaptive responses can be compensated for via glutamine metabolism bypass pathways or by circumventing glutamine metabolism, thereby rewiring them to survive and proliferate [211]. This process can, in turn, lead to an escalation in the risk of drug resistance and relapse. Therefore, a comprehensive understanding of these compensatory mechanisms is imperative for the development of more effective therapeutic strategies that target cancer cells [212].

### Cancer cells utilize glutamine metabolism-related branched pathways involved in the development of drug resistance

Glutamine synthetase (GS), an enzyme that functions at the origin of glutamine metabolism, is the sole regulatory factor that governs glutamine synthesis from its fundamental stages, thereby playing a pivotal role in the progression of cancer [213]. It has been demonstrated that a decrease in GS deficiency results in the inhibition of various signaling cascades associated with glutamine metabolism. In the event of a GS mutation, a multitude of additional amino acid biosynthetic pathways are known to undergo compensatory activation, encompassing arginine-proline, glycine-serine-threonine, and alanine-aspartate-glutamate metabolism. In instances where the citric acid cycle is blocked, a significant amount of intracellular glutamate is rerouted through transamination, an alternative metabolic pathway. Concurrently, pivotal metabolic enzymes within the amino acid synthesis pathway, including glutamate-oxaloacetate transaminase 1 (GOT1), glutamate-pyruvate transaminase 2 (GPT2), pyrrolidine-5-carboxylic acid reductase 1 (PYCR1), and phosphoserine aminotransferase 1 (PSAT1), demonstrated increased expression [214]. It has been demonstrated that when GS is inhibited, a variety of metabolic pathways other than glutamine synthesis are activated, thereby underscoring the remarkable plasticity of cancer cell metabolism. The utilization of stable isotope labeling of glutamine synthetase substrates was instrumental in elucidating its function in the starvation response of cancer cells. This approach revealed that the metabolic compensatory pathways that enable cells to overcome glutamine depletion are contingent on the capacity to synthesize glutamine via glutamine synthetase.

The downstream metabolite of glutamine, asparagine, plays a critical role in tumors. As indicated by the extant literature, asparagine synthetase (ASNS) catalyzes the biosynthesis of asparagine and glutamine [215]. As demonstrated in the extant literature, in circumstances where glutamine is depleted, tumor cells have been observed to circumvent glutamine metabolism, instead relying on asparagine to sustain their growth [216]. Inhibition of glutamine catabolism prompts cells to adopt an alternative pathway of catalytic activity involving ASNS, GAD (glutamic acid decarboxylase), and GABA (γ-aminobutyric acid) shunts. These shunts serve to mediate the link between glutamine and MTORC1 autophagy signaling [217]. The activation of the compensatory pathway enables cancer cells to survive, proliferate, and acquire drug resistance. ASNS metabolizes glutamine and, in combination with the GAD and GABA shunts, provides the cell with a backfill entry point in the TCA cycle that produces oxaloacetate and ATP in the absence of glutamine catabolism. In the event of GLS or ASNS inhibition, compensatory mechanisms may emerge, potentially accounting for the diminished anticancer efficacy observed in GLS targeting. In instances where ASNS plays a pivotal metabolic role, glutamine reprograms glutamine metabolism primarily through cancer cells with aberrant GAD1 expression to synthesize GABA, a neurotransmitter predominantly found in non-neural tissues. Elevated levels of GABA are indicative of a poor prognosis [218].

In instances where GDH is inhibited, aspartate aminotransferase 1 (GOT1) assumes a pivotal function in the dysregulation of glutamate metabolism. The process of transamination by GOT1 results in the generation of α-KG and aspartic acid (Asp), thereby contributing to the maintenance of the TCA cycle. Concurrently, the generated Asp is converted to oxaloacetic acid (OAA) by GOT1 in the cytoplasm. OAA is then converted to malic acid, which in turn generates NADPH through ME1. This process reduces the level of ROS, maintains the cellular redox balance, and protects the tumor cells from oxidative damage [219]. The GOT1-mediated glutamine metabolic pathway is a critical survival mechanism when the major glutamine pathway is inhibited, and it promotes tumorigenesis and development by replenishing the tricarboxylic acid (TCA) cycle, maintaining redox homeostasis, and providing the essential precursor, aspartic acid (Asp), for nucleotide production. Consequently, the targeted inhibition of GOT1, in conjunction with its upstream and downstream pathways, or the combined inhibition of the major metabolic pathways of glutamine, has emerged as a promising novel strategy to overcome metabolic therapy resistance.

### Cancer cells bypass glutamine metabolism involved in drug resistance

#### Glycolysis

In instances where the metabolic process of glutamine is impeded, glycolysis can emerge as a compensatory pathway, contributing to the emergence of drug resistance. Recent findings have indicated that the inhibition of glutamine metabolism in cancer cells results in an enhancement of glycolysis, a process that is facilitated by metabolic repair and immune evasion induced by up-regulated PD-L1 [220]. Additionally, the deprivation or inhibition of glutamine has been observed to shift the metabolic state of glioblastoma U87MG cells towards glycolysis, accompanied by an up-regulation of the expression of the stemness marker CD133 [221]. The serine de novo synthesis pathway (SSP) represents a pivotal metabolic bypass of glycolytic metabolism, whereby the glycolytic intermediate 3-phosphoglyceric acid is converted into serine and glycine through the action of numerous metabolic enzymes, including phosphoglycerate dehydrogenase (PHGDH). Phosphatidylserine aminotransferase 1 (PSAT1) and phosphatidylserine phosphohydrolase (PSPH) have been identified as key mediators of one-carbon metabolism. This process provides the material and energy basis for the rapid proliferation of cancer cells and maintains redox homeostasis in tumor cells. Recent findings have demonstrated that glutamine-addicted breast cancer cells exhibit a capacity to adapt to chronic glutamine starvation or GLS inhibition through AMPK-mediated upregulation of the serine synthesis pathway. This pathway is found to be highly dependent on SSP-provided α-KG when glutamine metabolism is inhibited [222]. Mechanistically, PSAT1 possesses the distinctive capacity to perpetually generate α-KG in the absence of glutamine. Consequently, the inhibition of SSP hinders the adaptation to glutamine blockade, thereby eliciting potent pharmacological synergies that impede the growth of breast tumors. In the study of triple-negative breast cancer (TNBC), researchers found that hybridized BLG@TPGS NPs were prepared by doping the multipath energy inhibitors berberine (BBR) and lonidamine (LND) as well as the chemotherapeutic agent garcinia cambogia (GA), which inherited the mitochondria-targeting ability of BBR and accurately accumulated in the mitochondria, then induced starvation therapy, which effectively eradicated cancer cells by coordinating the shutdown of tumor cells through the “three-tendency strategy” to cut off the mitochondrial respiration, glycolysis and glutamine metabolism [223]. A synergistic combination with chemotherapy has been demonstrated to extend inhibition of tumor proliferation and migration. Furthermore, it was demonstrated that exogenous pyruvate, functioning as a complementary substrate, impeded the reduction of fumarate under CB-839 treatment conditions. Furthermore, the endogenously produced and secreted pyruvate in TNBC cell lines exhibited the capacity to markedly diminish the sensitivity of recipient cells to glutaminase inhibition through a paracrine mechanism [224].

#### Fatty acid metabolism

It is also noteworthy that lipid metabolism can be involved in the development of drug resistance as a compensatory pathway. A study of TNBC cells revealed that those resistant to CB-839 exhibited increased levels of both the CPT2 protein and CPT1 activity. Consequently, TNBC cells resistant to CB-839 mobilized a greater amount of fatty acids into the mitochondria for oxidation. This phenomenon was associated with the AMPK and acetyl-CoA carboxylase signaling pathways. Furthermore, the dual inhibition of glutaminase and CPT1 has been shown to reduce the proliferation and migration of CB-839-resistant cells in comparison to the inhibition of individual enzymes. Consequently, the concurrent targeting of glutaminase and CPT1 activities may hold therapeutic potential for the management of CB-839-resistant tumors [225]. G protein-coupled receptors (GPCRs) represent a prevalent area of research in the pharmaceutical sciences, with these receptors activating diverse signaling pathways in response to various ligands and depending on the specific tissue type, as illustrated by the following examples: When targeting G-protein-coupled receptors (GPCRs), alternative pathways such as fatty acid β-oxidation (FAO) and other alternative pathways of glutamine metabolism provide nutrient support to cancer cells. Studies in different cancer models have shown that in vitro and in vivo experiments have reported the effects of FAO inhibitors on cancer inhibition. Co-targeting GPCR with glutamine metabolism will likely further inhibit the proliferation of cancer cells, contributing to the anticancer potential and overcoming drug resistance [226].

#### ATF4

When cancer cells sense glutamine deprivation or inhibition of glutamine metabolism, it causes cancer cell stress, triggering an increase in ATF4 transcription. ATF4 transcription plays a key role in maintaining cellular homeostasis, thereby promoting cellular adaptation. In a study on pancreatic cancer, CB-839 was utilized to target glutamine metabolism, resulting in the effective inhibition of cell growth in pancreatic cancer cells [227]. This inhibition occurred through the mechanism of phosphorylation of activated GCN2, a pivotal factor in initiating protein synthesis. EIF2α, the eIF2α subunit of eIF2, was subsequently activated, leading to the activation of the GCN2-ATF4 signaling pathway [228]. Researchers have identified elevated levels of ATF4 expression in GS-deficient cells, which has been shown to result in increased expression of aminoacyl-tRNA synthetase (ARS) [214]. Furthermore, ATF4, under conditions of stress, has been observed to interact with mTORC, thereby influencing cell survival. In the study by Kim et al., it was found that in cases where Gln is deficient or inhibited, the presence of oxidative stress in cancer cells can specifically activate the Hippo signaling pathway’s YAP protein. This activated YAP then promotes the transcriptional induction of ATF4, which plays a role in the expression of genes involved in amino acid homeostasis, including Sestrin2. The induction of Sestrin2 by YAP, in turn, affects cell survival by inhibiting the mTORC1 pathway [229]. An exhaustive investigation revealed that an augmentation in ATF4, emanating from the mTORC2-PKC-Nrf2 signaling pathway, culminates in the induction of ATF4 expression and the promotion of Sirt5, a pivotal transcriptional target of ATF4. Sirt5, a NAD-dependent desuccinylase, fosters cancer cell survival during metabolic stress. Further studies revealed that Sirt5 plays an important role in supporting cancer cell metabolism by regulating various enzyme activities and protecting glutaminase C (GAC), an enzyme essential for glutamine catabolism, from degradation. In addition, ectopic expression of Sirt5 compensates for the reduction of ATF4 due to glutamine deprivation-induced stress in stressed cells [230]. Furthermore, glutamine deprivation has been observed to augment ATF4-mediated one-carbon metabolism. Inhibition of the first rate-limiting enzyme of one-carbon metabolism (PHGDH) has been shown to promote cell growth arrest, a phenomenon that occurs as a result of an accumulation of intracellular reactive oxygen species (ROS) following the inhibition of glutamine metabolism. The co-suppression of glutamine and one-carbon metabolism has been demonstrated to enhance the anticancer effect of drugs utilized in patients diagnosed with undifferentiated thyroid cancer (ATC), while concurrently mitigating the development of drug resistance to a certain extent. It is evident that one-carbon metabolism has a potential role in regulating cell fate during metabolic stress and can be a potential therapeutic target for enhancing antitumor effects [231]. As would be expected, ATF4 plays a critical role in cellular adaptation to glutamine deprivation, and it can promote cellular adaptation by bypassing the glutamine metabolism-regulated signaling pathway. ATF4 and its downstream effects have emerged as promising therapeutic targets for cancer therapy. The efficacy of apatinib in the treatment of solid tumors, including non-small cell lung cancer (NSCLC), has been demonstrated. The combination of practical apatinib and ATF4 silencing has been shown to eliminate glutamine metabolism in NSCLC cells. In addition, the study demonstrated that the knockdown of ATF4 augmented the antitumor efficacy of apatinib in both in vitro and in vivo models [232]. It has been demonstrated that the inhibition of glutamine metabolism produces the onset of drug resistance. Therefore, the combination of inhibitors that target glutamine metabolism in conjunction with ATF4 at an early stage of the clinic improves the sensitivity of anticancer drugs and improves patient prognosis.

### Challenges in targeting glutamine metabolism for drug resistance

Cancer cells in which glutamine metabolism is inhibited also undergo metabolic adaptation through multiple pathways to ensure their survival and proliferation, which is a serious challenge for tumor resistance. In order to address the issue of therapeutic resistance driven by reprogramming of tumor glutamine metabolism, future studies must thoroughly examine the heterogeneity of glutamine dependence in different genetic backgrounds (e.g., KRAS-mutant vs. EGFR-mutant tumors) and further elucidate the dynamic metabolic features of drug-resistant cells. In addition, further exploration is necessary to identify the molecular switches of tumor cells that activate alternative metabolic pathways (e.g., Glycolysis or Fatty acid oxidation) in response to glutamine deprivation. This investigation should also include the regulatory mechanisms of tumor-immune intercellular metabolic competition (e.g., the enhancement of CAR-T cell function through glutamine deprivation). Concurrently, the field continues to grapple with substantial challenges. On the one hand, metabolically targeted drugs may induce toxicity due to the basal requirement of glutamine in normal tissues; on the other hand, tumor metabolic plasticity may lead to therapeutic escape, which calls for the development of more selective combinatorial strategies of inhibitors and blockade of compensatory pathways. Finally, different types of cell lines within a single cancer are heterogeneous, and the bypass pathway for metabolic adaptation mechanism is also different when inhibiting glutamine metabolism. Therefore, it is essential to accurately control and study the molecular mechanism of drug resistance in different types of cancer cell lines and to enhance the sensitivity of combination drugs.

## Conclusion

Glutamine is the most abundant amino acid in plasma, and its metabolism, as a central part of cancer metabolic reprogramming, provides energy support for cancer cells and promotes proliferation, invasion, metastasis, and immune escape. The elevated expression of critical transporter proteins and metabolic enzymes, including ASCT2 and GLS1, has been demonstrated to be associated with tumor progression and a poor prognosis. This suggests that these proteins may serve as valuable therapeutic targets. Inhibitors targeting glutamine metabolism have demonstrated significant antitumor potential by impeding energy supply, inducing oxidative stress, and activating immune response. However, their clinical application remains challenging due to metabolic heterogeneity, compensatory pathway activation, and immunosuppressive effects. Furthermore, cancer cells have been observed to circumvent glutamine metabolism inhibition, leading to drug resistance. This circumvention occurs through the activation of alternative metabolic pathways, including glycolysis, fatty acid oxidation, and ASNS shunting. Alternatively, cancer cells may rely on ATF4-mediated stress mechanisms to bypass these inhibitors. In the future, there is a need to combine multi-omics technology to analyze the metabolic heterogeneity, develop multiple inhibitors targeting the compensatory pathway, and improve the killing effect of cancer cells through the combination of glutamine metabolism inhibition with immune checkpoint blockade, CAR-T therapy, and other applications.

In summary, research on glutamine metabolism offers a novel approach to cancer treatment. However, to overcome the current limitations, a synergistic integration of fundamental and clinical research is imperative. This will lead to substantial advancements in cancer treatment outcomes, providing renewed hope for cancer patients.

## Data Availability

No datasets were generated or analysed during the current study.

## Abbreviations

Ala: Alanine
ALDH18A1: Aldehyde dehydrogenase 18 family member A1
AMPK: Adenosine 5’-monophosphate (AMP)-activated protein kinase
ANXA2: Annexin A2
aPDL1: Anti-PDL1 antibody
AREG: Amphiregulin
ARPC1A: Actin-related protein 2/3 complex subunit 1 A
ASCT2: Alanine-serine-cysteine transporter 2
ASNS: Asparagine synthetase
Asp: Aspartic acid
ATF4: Activating transcription factor 4
AZA: Azacytidine
BBR: Berberine
BC: Breast cancer
BCa: Bladder cancer
cAMP: Cyclic adenosine monophosphate
CAR-T: Chimeric antigen receptor-T
CD69: Cell adhesion molecule 69
CD8: Cytotoxic T Lymphocyte Antigen 8
cDC1: Type 1 conventional dendritic cells
CEMIP: Cell migration-induced hyaluronan-binding protein
circRNAs: Circular RNAs
c-MYC: Myelocytomatosis viral oncogene homolog
CQ: Chloroquine
CRC: Colorectal cancer
CSC: Cancer stem cells
CTGF: Connective tissue growth factor
CTL: Cytotoxic T lymphocyte
DON: 6-diazo-5-oxo-l-norleucine
ECM: Extracellular matrix
EGFR: Epidermal growth factor receptor
EMT: Epithelial-mesenchymal transition
EphA2: Erythropoietin-producing hepatocellular A2
ERK: Extracellular regulated protein kinases
ESCC: Esophageal squamous cell carcinoma
FA-DCNP: Folic acid-targeted nanoparticles
FAO: Fatty acid β-oxidation
FAs: Fatty acids
FLIP: FLICE-inhibitory protein
GABA: γ-aminobutyric acid
GAD: Glutamic acid decarboxylase
GBM: Glioblastoma
GC: Gastric carcinoma
GCN5: General control non-derepressible 5
GDH: Glutamine dehydrogenase
Gln: Glutamine
GLS: Glutaminase
Glu: Glutamic acid
GLUD: Glutamate dehydrogenase
GLUL: Glutamate-ammonia ligase
GLULH: Glutathione
GLUT1: Glucose transporter 1
GMII: Glutamine metabolism immunity index
GOT1: Glutamate-oxaloacetate transaminase 1
GPCRs: G protein-coupled receptors
GS: Glutamine synthetase
Gsk3β: Glycogen synthase kinase 3β
GZMB: Granzyme B
HCC: Hepatocellular carcinoma
HDAC: Histone deacetylase
HEPG: Hepatocellular carcinoma
HNSCC: Head and neck squamous cell carcinoma
hSPAR: Human SPAR
ICB: Immune checkpoint blockade
IFN-β: Interferon-β
IL-1β: Interleukin-1β
IL-6: Interleukin-6
JAK: Janus kinase
Keap1: Kelch-like ECH-associated protein 1
LAT2: L-amino acid transporter protein 2
LATS: Large tumor suppressor kinase
LDHA: Lactate dehydrogenase
LNCaP: Human prostate cancer cells
lncRNAs: Long-stranded non-coding RNAs
LND: Lonidamine
LPO: Lipid peroxidation
LUAD: Lung adenocarcinoma
M1-TAMs: M1-type macrophages
M2-TAMs: M2-type macrophages
MDS: Myelodysplastic syndromes
MM: Multiple myeloma
MoS2: Molybdenum disulfide
MRPL35: Mitochondrial ribosomal protein L35
MSO: l-methionine sulfoximine
mTORC1: Mechanistic target of rapamycin complex 1
MZF1: Myeloid zinc finger 1
NADH: Nicotinamide adenine dinucleotide
NADPH: Nicotinamide adenine dinucleotide phosphate hydrogen
ncRNAs: Non-coding RNAs
NetG1: Netrin G1
NF-κB: Nuclear factor kappa-B
NKT: Natural killer T
NSCLC: Non-small cell lung cancer
NSUN2: NOP2/Sun RNA methyltransferase 2
OA: Oleic acid
OAA: Oxaloacetic acid
OC: Ovarian cancer
OSCC: Oral squamous cell carcinoma
OTUD4: OTU deubiquitinase 4
OXPHOS: Oxidative phosphorylation
PC: Pancreatic cancer
PCa: Prostate cancer
PDAC: Pancreatic ductal adenocarcinoma
PHGDH: Phosphoglycerate dehydrogenase
PKA: Protein kinase A
PM: Particulate matter
PRF1: Perforin 1
PSAT1: Phosphatidylserine aminotransferase 1
PTC: Papillary thyroid carcinoma
PYCR1: Pyrroline-5-carboxylic acid reductase 1
RA: Retinoic acid
ROS: Reactive oxygen species
rRNA: ribosomal RNA
S100A2: S100calcium-binding protein A2
SCLC: Small cell lung cancer
SIRT4: Sirtuin 4
SLC1A5-AS: SLC1A5 antisense long non-coding RNA
SLC38A2: Solute carrier family 38 member 2
SREBP-1: Sterol regulatory element binding transcription factor 1
SSP: Synthesis pathway
STAB1: SATB homeobox 1
STAT3: Signal transducer and activator of transcription
TARBP1: TAR (HIV-1) RNA-binding protein 1
TAZ: Transcriptional coactivator with PDZ-binding motif
TC2N: Tandem C2 structural domain nuclear protein
TCA: Tricarboxylic acid cycle
TEAD: TEA domain transcription factor
TGM2: Glutamine transaminase 2
TME: Tumour microenvironment
TNBC: Triple-negative breast cancer
TRAIL-R2: TNF-related apoptosis-inducing ligand receptor 2
tRNA: Transfer RNA
TrxR: Thioredoxin reductase
UPS: Ubiquitin-proteasome system
YAP: Yes-associated protein
α-KG: α-Ketoglutarate
